# Functional characterization of age-dependent *p16* epimutation reveals biological drivers and therapeutic targets for colorectal cancer

**DOI:** 10.1186/s13046-023-02689-y

**Published:** 2023-05-04

**Authors:** Li Yang, Xiaomin Chen, Christy Lee, Jiejun Shi, Emily B. Lawrence, Lanjing Zhang, Yumei Li, Nan Gao, Sung Yun Jung, Chad J. Creighton, Jingyi Jessica Li, Ya Cui, Sumimasa Arimura, Yunping Lei, Wei Li, Lanlan Shen

**Affiliations:** 1 USDA Children’s Nutrition Research Center, Department of Pediatrics, Baylor College of Medicine, TX Houston, USA; 2 Department of Statistics, University of California, Los Angeles, CA USA; 3 Division of Computational Biomedicine, Department of Biological Chemistry, School of Medicine, University of California, Irvine, CA USA; 4 Present address: Department of General Surgery, Shanghai Tongji Hospital, School of Life Sciences and Technology, Tongji University, Shanghai, China; 5 Department of Pathology, Princeton Medical Center, Plainsboro, NJ USA; 6 Department of Chemical Biology, Earnest Mario School of Pharmacy, Rutgers University, Piscataway, NJ USA; 7 Human Genome Sequencing Center, Baylor College of Medicine, Houston, TX USA; 8 Department of Biological Sciences, Rutgers University, Newark, NJ USA; 9 Department of Biochemistry, Baylor College of Medicine, Houston, TX USA; 10 Department of Medicine and Dan L Duncan Comprehensive Cancer Center, Baylor College of Medicine, Houston, TX USA; 11 Department of Medicine and Section of Gastroenterology and Hepatology, Baylor College of Medicine, Houston, TX USA; 12 Center for Precision Environmental Health, Department of Molecular and Cellular Biology, Baylor College of Medicine, Houston, TX USA

**Keywords:** Colon cancer, *p16* epimutation, Tumor microenvironment, Epigenetic and immunotherapy

## Abstract

**Background:**

Methylation of the *p16* promoter resulting in epigenetic gene silencing—known as *p16* epimutation—is frequently found in human colorectal cancer and is also common in normal-appearing colonic mucosa of aging individuals. Thus, to improve clinical care of colorectal cancer (CRC) patients, we explored the role of age-related *p16* epimutation in intestinal tumorigenesis.

**Methods:**

We established a mouse model that replicates two common genetic and epigenetic events observed in human CRCs: *Apc* mutation and *p16* epimutation. We conducted long-term survival and histological analysis of tumor development and progression. Colonic epithelial cells and tumors were collected from mice and analyzed by RNA sequencing (RNA-seq), quantitative PCR, and flow cytometry. We performed single-cell RNA sequencing (scRNA-seq) to characterize tumor-infiltrating immune cells throughout tumor progression. We tested whether anti-PD-L1 immunotherapy affects overall survival of tumor-bearing mice and whether inhibition of both epigenetic regulation and immune checkpoint is more efficacious.

**Results:**

Mice carrying combined *Apc* mutation and *p16* epimutation had significantly shortened survival and increased tumor growth compared to those with *Apc* mutation only. Intriguingly, colon tumors with *p16* epimutation exhibited an activated interferon pathway, increased expression of programmed death-ligand 1 (*Pdl1*), and enhanced infiltration of immune cells. scRNA-seq further revealed the presence of *Foxp3*^+^ Tregs and γδT17 cells, which contribute to an immunosuppressive tumor microenvironment (TME). Furthermore, we showed that a combined therapy using an inhibitor of DNA methylation and a PD-L1 immune checkpoint inhibitor is more effective for improving survival in tumor-bearing mice than blockade of either pathway alone.

**Conclusions:**

Our study demonstrated that age-dependent *p16* epimutation creates a permissive microenvironment for malignant transformation of polyps to colon cancer. Our findings provide a mechanistic rationale for future targeted therapy in patients with *p16* epimutation.

**Supplementary Information:**

The online version contains supplementary material available at 10.1186/s13046-023-02689-y.

## Introduction

Although an extensive catalog of DNA methylation alterations has been detected in patients with colorectal cancer (CRC), elucidating the functional contributions of these modifications during tumor initiation and maintenance remains an important unmet need. Mitotically stable gene silencing resulting from epigenetic alteration of promoter DNA methylation, known as epimutation, was proposed by Robin Holliday [1] as one possible mechanism for loss of tumor suppressor function in Knudson’s classic two-hit gene inactivation model [2]. Importantly, unlike genetic mutations, epigenetic mechanisms are intrinsically malleable and thus represent attractive therapeutic targets for improving clinical care of CRC patients [3, 4]. To this end, we seek to better understand the mechanisms by which epimutations frequently observed in cancer cells contribute to carcinogenesis.

Epimutation of the cyclin-dependent kinase inhibitor 2A (*CDKN2A*) gene, also known as *p16*, is among the most common epigenetic events in human CRCs [5, 6], and this modification is frequently detected in preneoplastic lesions [7, 8]. Indeed, *p16* epimutation originates in normal colon tissues, where it occurs as a function of aging, a phenomenon that is conserved in both humans and mice [9, 10]. Notably, such age-associated epimutations are preferentially located on CpG islands marked with polycomb complex [10] and *p16,* which controls cell cycle progression, promotes cellular senescence, and is one of the best documented polycomb-bound genes [11, 12]. Prompted by these observations, we created a mouse model of engineered *p16* promoter hypermethylation, which leads to accelerated *p16* epimutation in somatic tissues during aging and predisposes mice to spontaneous tumor development [13]. This established mouse model of *p16* epimutation enables us to answer clinically relevant questions that cannot easily be addressed using in vitro systems. Of particular interest, we can determine whether *p16* epimutation drives malignant progression of intestinal tumors and if reversal of these epigenetic defects in *p16* suppresses tumor growth.

Many cancers arise through successive accumulation of genetic and epigenetic alterations that collectively drive disease progression and metastasis [14]. In this regard, genetically engineered mouse models have provided direct evidence that combined mutations can accelerate the growth of intestinal tumors and promote the development of a malignant phenotype. Published studies have established such a connection between *p16* inactivation and driver gene mutations (*e.g*., in *Apc* and *Braf*) in intestinal tumorigenesis models [15–17]. However, these studies have only focused on investigating the functional effect of *p16* gene mutations, which rarely occur in human CRCs. In addition, most mouse studies used young animals of 6 − 8 weeks of age, which is equivalent to approximately 15 − 20-year-old humans. As a result, many of the biological processes that underlie the age-dependency of cancer were not considered.

Here, to determine whether age-dependent *p16* epimutation promotes intestinal carcinogenesis in combination with *Apc* mutation, we generated a mouse model that replicates these two events, which are commonly observed in human CRC [18]. Using this model, we found that *p16* epimutation promotes the malignant transformation of intestinal tumors initiated by *Apc* mutation. In addition, colon tumors with combined *Apc* mutation and *p16* epimutation exhibit a remarkable immune phenotype with high levels of interferon signaling and programmed death-ligand 1 (*Pdl1*) expression. Single-cell RNA sequencing (scRNA-seq) analysis further revealed dynamic changes in the tumor microenvironment (TME) in *Apc*-mutant mice with *p16* epimutation, identifying distinct immune cell subpopulations that contribute to T cell dysfunction and tumor immune evasion. Lastly, we found that a combined therapy involving inhibition of both DNA methylation and the PD-L1 immune checkpoint improves survival in our mouse model of CRC, suggesting that such combined therapies may hold clinical promises for CRC patients with *p16* epimutation.

## Materials and methods

### Experimental animals

The mouse lines used in this study have been described previously; in brief, *p16*^*cis/cis*^ mice [13] were created by targeted knock-in of a 140-bp DNA sequence (*cis*-element) that facilitates spread of DNA methylation at the *p16* promoter in *cis*. *Apc*^*Min/*+^ mice [19] carrying a heterozygous germline mutation at codon 850 of the *Apc* gene were crossed with *p16*^*cis/cis*^ mice to generate *Apc*^*Min/*+^; *p16*^*cis/cis*^ mice. All mice were on a C57BL/6 background. Details for PCR genotyping assays, including primer sequences and PCR conditions, are summarized in Supplementary Table 1. All animal research was performed in accordance with the NIH Guide for Care and Use of Laboratory Animals and approved by the Baylor College of Medicine Animal Care and Use Committee. Mice were monitored bi-weekly by determining body weight measurements and assessing rectal prolapse size. We used the following criteria for early euthanasia: > 20% weight loss, progression of rectal prolapse to greater than 3 mm of tissue protrusion, and/or severe signs of a moribund condition.

### Cell culture

Primary MEFs (*cis*-MEFs) were isolated from *p16*^*cis/cis*^ mice at embryonic day 12.5 (E12.5) using the Pierce™ Mouse Embryonic Fibroblast Isolation Kit (Thermo Fisher Scientific, Waltham, MA, USA). As a control, we isolated MEFs (ctr-MEFs) from mice carrying a control element knocked into the same targeted site, as previously described [13]. MEFs were cultured in Dulbecco’s Modified Eagle Medium (DMEM; Gibco, Thermo Fisher Scientific), containing 10% fetal bovine serum (FBS; R & D Systems, Minneapolis, MN, USA), 2-mM L-glutamine (Gibco, Thermo Fisher Scientific), and 1% penicillin/streptomycin (Invitrogen, Thermo Fisher Scientific). To remove the *cis*-element, we infected *cis*-MEFs at passage 19 with adenovirus Ad5CMVCre (Gene Vector Core, Baylor College of Medicine, Houston, TX, USA). After PCR validation to ensure complete excision of the *cis*-element, MEFs were exposed to DAC (Sigma-Aldrich, St. Louis, MO, USA) at varying concentrations (0.2, 0.5, or 1 μM) or phosphate-buffered saline (PBS) for 72 h, as previously described [20].

### Whole-genome bisulfite sequencing (WGBS) analysis

WGBS was performed and analyzed as previously described [21, 22]. Briefly, we used 500 ng genomic DNA from primary MEFs, i.e., ctr-MEF at passage 7 and *cis*-MEF at passage 12. Sonicated, adaptor-ligated DNA was treated with sodium bisulfite by the EZ DNA Methylation-Direct kit (Zymo Research). The bisulfite modified DNA was amplified (18 cycles) using adaptor-specific primers and fragments of 200–500 bp were isolated. The quantity and size distribution of libraries were determined using the Pico Green fluorescence assay and the Agilent 2100 Bioanalyzer, respectively. Each library was sequenced as 150 bp paired end reads with planned 30 × coverage per sample. The reads were mapped to mouse genome (mm10) using BSMAP with default parameters [23]. Using the “CAMDA” function in “CAMDA.py” toolkit^*47*^, the average methylation ratio of each CpG was calculated as the number of unconverted CpGs divided by the total number of read covering that CpG. All WGBS data been uploaded to GEO and are available at the accession number GSE214032.

### CRISPR-mediated targeted demethylation

We used the dCas9-SunTagTET1 system [24] to perform targeted *p16* promoter demethylation. Plasmids containing *p16*-specific gRNA or non-targeting control gRNA were constructed using an all-in-one vector from Addgene (#82,559; Watertown, MA, USA) and Gibson Assembly (NEB, Ipswich, MA, USA). gRNA sequences are listed in Supplementary Table 2. Transfection of *cis*-MEFs was performed with equimolar amounts of plasmid, using Lipofectamine 20,000 Reagent (Life Technologies, Thermo Fisher Scientific), according to the manufacturer’s instructions. The dCas9-SunTag system contains the scFv-GFP-TET1CD fusion protein which enables FACS sorting to isolate vector-expressing cells. At 48 h post-transfection, MEFs were sorted using a FACS Aria Fusion flow cytometer (BD Biosciences, Franklin Lakes, NJ, USA) to isolate GFP-positive cells for *p16* methylation and expression analyses.

### DNA methylation and gene expression analysis

Quantitative bisulfite-pyrosequencing analyses to measure DNA methylation was performed as previously described [13, 25]. Bisulfite sequencing of cloned PCR products was used to confirm methylation of CpG sites. Primer sequences and sequencing assays are listed in Supplementary Table 3. Quantitative qRT-PCR was performed to measure mouse *p16* and ERV expression levels as previously described [13, 26]. Assay designs are summarized in Supplementary Table 4. All experiments were carried out in triplicate and relative gene expression was normalized to *β-actin* expression on an ABI Step OnePlus Detection System.

### Histology

For histological analyses, mouse intestines and tumors were fixed in 10% neutral buffered formalin. Fixed tissues were paraffin-embedded, sectioned, and stained with H&E, according to standard laboratory protocols at the Cellular and Molecular Morphology Core at the Texas Medical Center Digestive Diseases Center.

### RNA-seq analysis

RNA-seq was performed using 1-μg RNA extracted from colonic crypts, as previously described [22, 27]. Prior to sequencing, RNA was subjected to quality control analysis using an Agilent 2100 Bioanalyzer with the Agilent RNA 6000 Nano Kit (Agilent Technologies, Santa Clara, CA, USA). RNA-seq libraries were prepared using standard BGI protocols (mRNA enrichment by rRNA depletion and oligo dT selection) and sequenced on the BGISEQ-500 platform (BGI Group, Shenzhen, China), with a planned sequencing depth of 25 million reads per sample. BOWTIE2 software [28] was used for efficient realignment of RNA sequences, and gene expression levels for each sample were calculated with RSEM [29]. For DEG analysis, we used DEseq2 [30] and ranked genes by fold change (> 2) and adjusted *P*-value based on multiple testing correction (Bonferroni). All RNA-seq data have been uploaded to GEO and are available at the accession number GSE213568.

### Microsatellite instability (MSI) analysis by fluorescent PCR

Five microsatellite loci were analyzed in colon tumors from *Apc*^*Min/*+^ and *Apc*^*Min/*+^; *p16*^*cis/cis*^ mice, including three mononucleotide (BAT-24, BAT-59, and BAT-67) and two dinucleotide markers (DiMit79 and TG27) based on the published recommendations [16, 31, 32]. PCR primers and fluorescent labeling are summarized in Supplementary Table 5. For PCR amplifications, we used DNA isolated from colon tumors as well as healthy tissues (liver). PCR fragments were separated on a 3730 DNA analyzer (Applied Biosystems) following the manufacture’s protocol and the raw data were analyzed with GeneMapper 5 software. If the profiles of all markers are identical to those seen in normal tissues, the tumor is classified as microsatellite stable (MSS).

### Tumor preparation and flow cytometry

Colon tumors were dissected, cut into small pieces, and further dissociated into a single-cell suspension using the Mouse Tumor Dissociation Kit (Miltenyi Biotec, Bergisch Gladback, North Rhine-Westphalia, Germany) and a gentleMACS™ Octo Dissociator (Miltenyi Biotech), according to the manufacturer’s instructions. Digested tumors were filtered through 70 μM filters, washed with PBS, and then subjected to flow cytometry analysis. For each sample, red blood cells were removed using Red Blood Cell Lysis Solution (Miltenyi Biotech), and the remaining cells were treated with TruStain FcX™ PLUS blocking solution (BioLegend, San Diego, CA, USA). Cells were then analyzed with the Zombie Yellow Fixable Viability Kit (BioLegend) for live/dead staining and labelled using the following antibodies: PE/Dazzle™ 594 anti-CD45 (clone 30-F11), APC anti-CD3 (clone 17A2), APC/Cy7 anti-CD4 (clone RM4-5), Alexa Fluor® 700 anti-CD8a (clone 53–6.7), Brilliat Violet 421™ anti-CD11b (clone M1/70), PE anti-Ly6G/Ly6C (Gr-1) (clone RB6-8C5), all of which were purchased from BioLegend. Samples were acquired on a BD FACSymphony A5 High-Parameter Cell Analyzer (BD Biosciences).

### scRNA-seq

Colon tumors and adjacent normal tissues were collected and processed on the same day for scRNA-seq. Single-cell suspensions were prepared from tumor samples and normal colonic mucosa using the Tumor Dissociation Kit (Miltenyi Biotec) or with gentle TrypLE (Thermo Fisher Scientific) dissociation, respectively. Single live cells were isolated by flow cytometry, and 3’ gene expression libraries were generated using the Chromium Next GEM Single Cell 3’ Kit v3.1 (10 × Genomics, Pleasanton, CA, USA). These were then subjected to scRNA-seq as described previously [33]. In brief, single cells, reverse transcription (RT) reagents, gel beads containing barcoded oligonucleotides, and oil were loaded on a Chromium controller (10 × Genomics) to generate single-cell Gel Bead-in-Emulsions (GEMS) on which full length cDNA was synthesized and barcoded for each single cell. GEMS were then broken, and cDNAs from each single cell were pooled, cleaned up using Dynabeads MyOne Silane Beads (Invitrogen, Thermo Fisher Scientific), and amplified by PCR. The amplified products were then fragmented to an optimal size prior to end-repair, A-tailing, and adaptor ligation. The final library was sequenced on the Illumina Novaseq 6000 platform. All scRNA-seq data have been uploaded to GEO and are available at the accession number GSE213568.

### scRNA-seq data analysis

We used Seurat v4.0 [34] for scRNA-seq data processing and analysis. Cells were filtered to include only those with < 5% mitochondrial DNA, RNA counts between 250 and 30,000, and 250 to 5000 features. Doublets were removed using DoubletFinder [35], according to a recent benchmarking paper [36]. The homotypic proportion was estimated through unsupervised clustering using the Leiden algorithm [37]. To eliminate batch effects, MNNCorrect [38] and Seurat integration [34] were applied separately to the dataset with default parameters. MNNCorrect successfully grouped cell types together but left significant batch effects within clusters. Seurat integration grouped cell types together and successfully mixed batches. The remaining analysis was performed using the results from Seurat integration.

Optimal Uniform Manifold Approximation and Projection (UMAP) [39] parameters were determined using scDEED (manuscript under submission). Briefly, integrated data were clustered using the Leiden algorithm with resolution 2.5. Cells were annotated manually using markers found through Seurat’s FindAllMarkers. Note that besides the Leiden algorithm, the data were also clustered and annotated using the Louvain algorithm [40] at the same resolution. Based on marker analysis, some Louvain clusters contained multiple cell types, whereas the Leiden clusters displayed a more homogenous composition. Leiden clustering results were therefore used for final annotations and further analysis. Clusters with multiple unlikely cell type signatures and high ranking pANN scores from DoubletFinder (ranked in their original batch) were also removed.

DEGs were determined using the Wilcoxon Rank–Sum Test, MAST [41], and Clipper [42]. Analysis was restricted to genes with a log-fold change of at least 0.25 across two conditions. Correction for multiple testing in the Wilcoxon Rank–Sum Test and MAST was performed with the Benjamini–Hochberg method. Only genes found to be significant by all three methods were reported as DEGs. GO and Kyoto Encyclopedia of Genes and Genomes (KEGG) analysis was performed with ClusterProfiler [43].

### Drug treatments

We began drug treatments in *Apc*^*Min/*+^; *p16*^*cis/cis*^ mice at 12–15 weeks of age, when intestinal tumors had been established. For single-agent therapy, mice were randomly assigned to receive either 250 μg anti-PD-L1 (clone 10F.9G2, BioXCell) or the same amount of IgG2b isotype control (clone LTF-2, BioXCell). Treatments were administered by i.p. injection every 3 days, for a total of six treatments. For combination therapy, mice were treated with DAC (1.0 mg/kg, i.p.) on Monday and anti-PD-L1 or control IgG2b on Thursday for 6 or 10 consecutive weeks in females and males, respectively.

### 3D tumor organoid culture

Colonic tumor organoids were cultured as described previously with some modifications [44]. Briefly, colon tumor tissues were minced and enzymatically digested with 1-mg/ml collagenase type IV (Gibco, Thermo Fisher Scientific) for 30 min at 37 °C, with intermittent shaking. Digested cell fragments were washed with cold washing buffer, containing Ham's F-12 Nutrient Mix, 5% FBS, 15-Mm HEPES, 2.5-μM Rock-inhibitor Y-27632, 2-mM L-glutamine, 1% penicillin/streptomycin, 0.25-µg/mL amphotericin B, 50-µg/mL gentamicin, and 100-μg/ml Primocin™, and then filtered through a 70-μm cell strainer. Dissociated cells were suspended in Matrigel® Matrix (Corning Inc, Corning, NY, USA) at equal amounts and plated in pre-warmed 24-well plates with WRNE culture medium, containing Wnt-3A, R-spondin 3, and Noggin. Media was changed every 3 days, and cells were passaged after 5–7 days. To enrich for tumorigenic organoids, after two passages, organoids were grown and maintained in NE medium, without Wnt-3A and R-spondin. For DAC treatment, organoids were exposed to various concentrations of DAC, ranging from 0.2 to 2 μM. Cell growth was monitored using the CellTiter-Glo 3D Cell Viability Assay (Promega, Madison, WI, USA) based on the amount of ATP present, according to the manufacturer’s instructions. Luminescence was detected with a GloMax Discover Microplate Reader (Promega).

### Statistical analysis

All statistical analyses were performed using GraphPad Prism v6. Quantitative DNA methylation and expression results are expressed as the mean ± standard error of the mean (SEM). Significance was determined using the two-tailed Student’s *t*-test or one way ANOVA of more than two groups, and *P* < 0.05 was considered statistically significant.

## Results

### Engineered *p16* epimutation recapitulates key features of age-associated epigenetic silencing

**Fig. 1:**
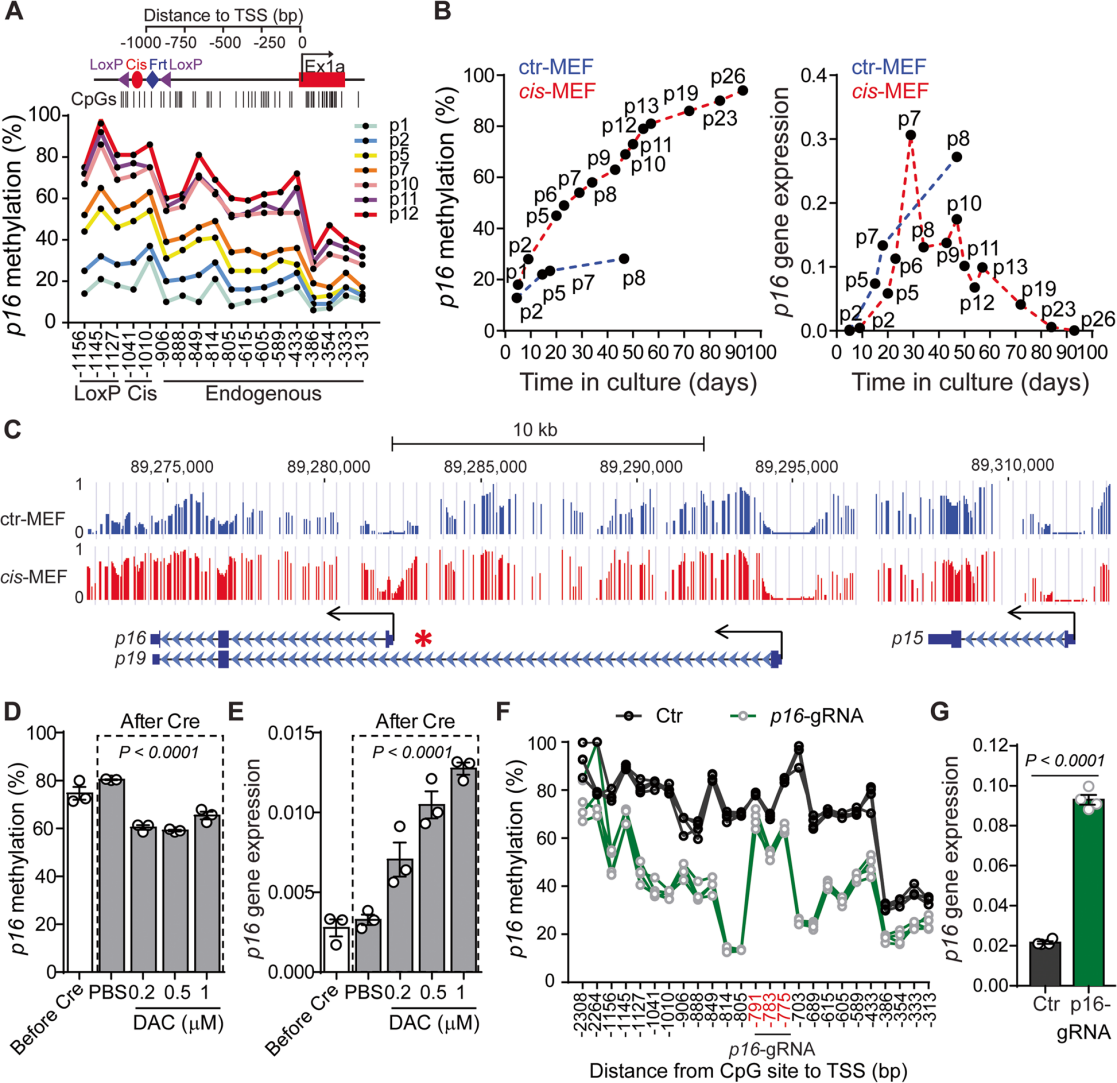
Engineered *p16* epimutation recapitulates key features of age-associated epigenetic silencing. **A** *p16* DNA methylation profiles in *cis*-MEF following serial passaging (p). A schematic of the *p16* promoter of the *cis*-element knock-in allele with the CpG maps is shown. **B** Kinetics of *p16* promoter methylation and mRNA expression in ctr-MEF and *cis*-MEF. Methylation levels were averaged from CpGs from − 814 bp to − 589 bp relative to the TSS. Note that *cis*-MEF cells grew for more than 25 passages; in contrast, the controls (ctr-MEF) entered growth arrest at passage 8 (p8) via *p16* up-regulation. The *p16* gene expression is relative to *β–actin.* **C** UCSC Genome Browser tracks showing the DNA methylation status of *p16*, *p19*, and *p15* in the INK4/ARF locus on chromosome 4. The chromosomal coordinates are annotated on the top. The WGBS tracks show DNA methylation profiling in ctr-MEF (p7) and cis-MEF (p10). The height of each bar represents the methylation level of an individual CpG between 0 and 1 (100%). Promoters are indicated by angled arrows, and the knock-in location is indicated by an asterisk. **D** Comparisons of *p16* promoter methylation in *cis*-MEF before and after Ad-Cre mediated *cis*-element excision with increasing doses of DAC. The culture passage number of *cis*-MEF is at p23. **E** Side-by-side comparisons of *p16* expression in *cis*-MEF under the same conditions as (**D**). For (**D)** and (**E**), data are shown as mean ± SEM with individual values from three independent experiments. *P* values were determined by a one-way ANOVA test. **F** dCas9-SunTagTET1-mediated targeted demethylation of *p16* in *cis*-MEF. *p16*-gRNA indicates GFP-positive cells treated with a *p16*-specific gRNA that binds the promoter region from − 792 bp to − 769 bp relative to the TSS. Ctr indicates GFP-negative cells without targeted demethylation. Data are shown from three independent experiments. **G** Targeted promoter demethylation resulted in *p16* gene reactivation. The culture passage number of *cis*-MEF is at p23. *P* values were determined by a two-tailed Student’s *t*-test

We previously generated a mouse model of *p16* epimutation through targeted knock-in of a 140-bp *Alu*-related DNA sequence (*cis*-element) that facilitates the spread of DNA methylation at promoter CpG islands (CGIs) in *cis* [13, 20]. Here, to validate our approach for modeling age-associated epigenetic events, we generated primary mouse embryonic fibroblasts (MEFs) from *cis*- or control (ctr)-element knock-in mice. We mapped DNA methylation patterns in an approximately 1 kb region at *p16* promoter and determined whether engineered *p16* epimutation leads to spontaneous immortalization of primary MEFs resulting from loss of *p16* function. Indeed, as measured during cell passaging, we observed initial methylation seeding within the *cis-*element, followed by increased methylation gradually spreading toward the endogenous *p16* promoter at later passages (Fig. 1A). Consistent with these observations, this increase in *p16* promoter methylation results in transcriptional suppression of *p16* in *cis*-element knock-in MEFs (*cis*-MEF), and these cells can be expanded well beyond the senescence checkpoint (*i.e*., at eight passages when ctr-MEFs are senescent; Fig. 1B). Furthermore, using whole genome bisulfite sequencing (WGBS) for a comprehensive DNA methylation profiling, we showed that *cis*-element-mediated approach specifically induces *p16* promoter hypermethylation; for example, the neighboring *p19*^*Arf*^ (*p14*^*Arf*^ in humans) and *p15*^*Ink4b*^ promoters, located > 11 kb and > 27 kb away, respectively, were not affected (Fig. 1C).

Next, to rule out the possibility that *p16* methylation is secondary to gene silencing (*e*.*g*., the *cis*-element recruits other silencers), we used adenovirus-Cre (Ad5CMVCre, referred to hereafter as Ad-Cre) to remove the *loxP*-flanked *cis*-element in *cis-*MEFs. We found that both *p16* promoter methylation (Fig. 1D) and gene silencing (Fig. 1E) are stably maintained, even two months after *cis*-element removal. In addition, treatment with the hypomethylating agent 5-aza-2’-deoxycytidine (DAC) restores *p16* expression in a dose-dependent manner. We then applied the CRISPR method [24] and used the dCas9-SunTagTET1 system to selectively demethylate DNA at the *p16* promoter. Remarkably, we found that one *p16*-specific sgRNA (*p16*-gRNA) induces greater demethylation at neighboring CpGs (*i.e*., within 200 bp of the *p16*-gRNA target site) than treatment with DAC at the standard dose of 0.5 µM (Fig. 1F). Interestingly, methylation levels at CpGs within the *p16*-gRNA-binding site are unchanged in *p16-*gRNA-transfected MEFs, perhaps because they are inaccessible to the TET1 demethylase. Nevertheless, selective CRISPR-mediated promoter demethylation also induces *p16* expression (Fig. 1G). These results demonstrate that our approach successfully models *p16* epimutation in which the *p16* gene activity is directly controlled by the engineered promoter methylation.

### *p16* epimutation cooperates with *Apc* mutation to drive adenoma–carcinoma progression

**Fig. 2:**
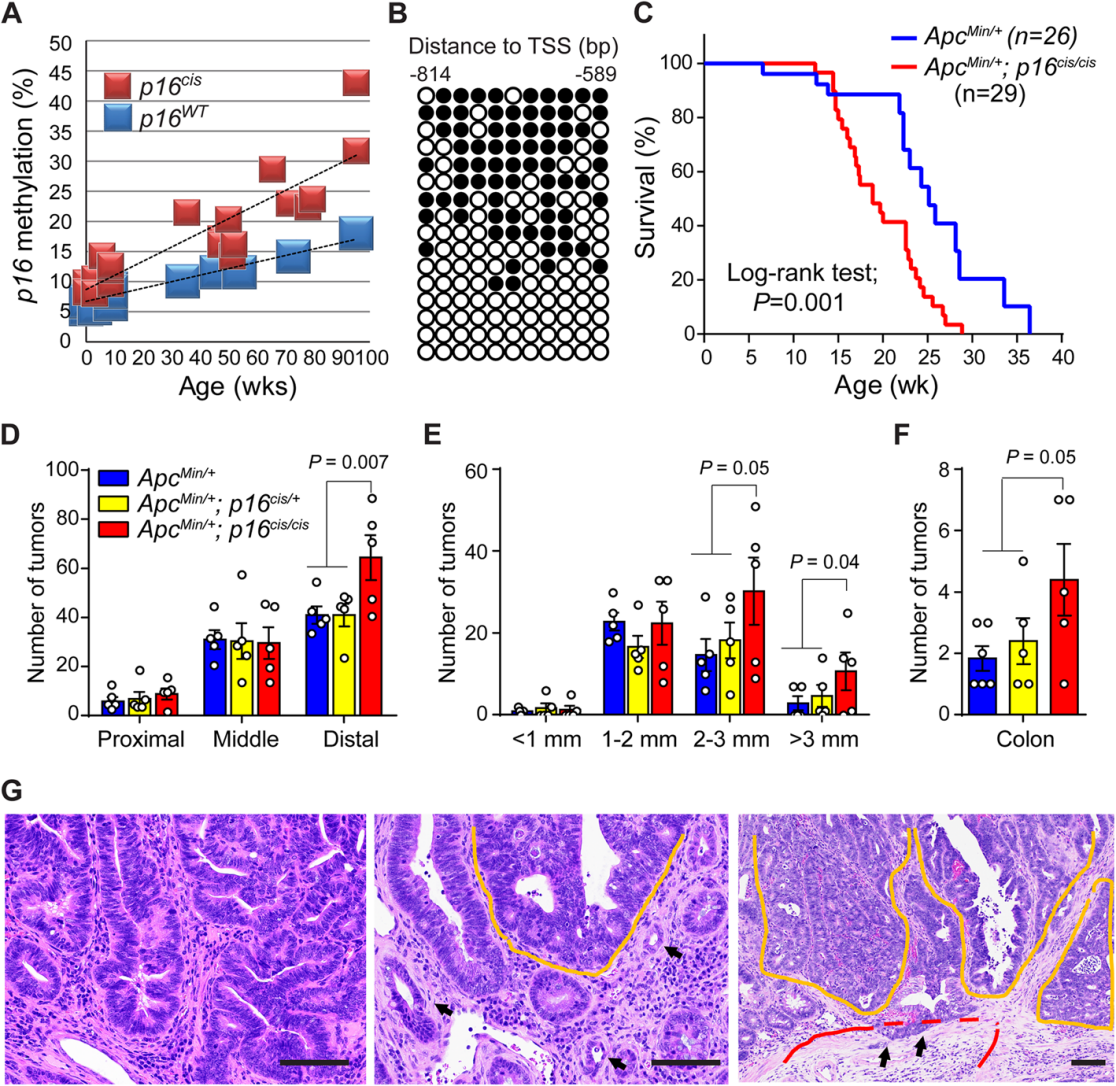
Age-dependent *p16* epimutation cooperates with *Apc* mutation to promote colon cancer. **A** Accelerated methylation of *p16*^*cis*^ alleles compared with *WT* alleles in intestinal tissues during aging as determined by bisulfite pyrosequencing. **B** Bisulfite PCR, cloning, and sequencing show a clonal expansion pattern associated with *p16* promoter hypermethylation in aged intestines. Each row represents a single sequenced molecule. Black and white circles represent methylated and unmethylated CpGs, respectively. **C** The addition of *p16* epimutation in *Apc*-mutant mice shortened survival. Survival was compared using the Kaplan–Meier method in *Apc*^*Min/*+^ mice (10 males and 16 females) and *Apc*^*Min/*+^*; p16*^*cis/cis*^ mice (15 males and 14 females). **D** At 15 wk of age, *Apc*^*Min/*+^; *p16*^*cis/cis*^ mice developed more tumors in the distal small intestines compared to *Apc*^*Min/*+^ and *Apc*^*Min/*+^; *p16*^*cis/*+^ mice. **E** Tumor number vs. size in the distal small intestines. **F** More colon tumors were found in the *Apc*^*Min/*+^; *p16*^*cis/cis*^ mice at 15 wk of age compared to the other two groups. For figures (**D**–**F**), data are mean ± SEM with individual values. *P* values were determined by a two-tailed Student’s *t*-test. **G** H&E staining of the colon tumor sections revealed histologic features of malignant transformation in *Apc*^*Min/*+^; *p16*^*cis/cis*^ mice. Yellow lines indicate tubular adenomas with extensive high-grade dysplasia, and arrows indicate focally invasive adenocarcinoma. Scale bars: 100 μm

To further characterize the effects of engineered *p16* epimutation in vivo, we focused on the intestine which represents the most rapidly renewing tissue. To assess changes in DNA methylation during normal aging, we generated *p16*^*cis/*+^ mice carrying the *cis*-element knock-in at one allele and used forward primers specific for knock-in or WT sequences to measure *p16* promoter methylation at each allele separately. As shown in Fig. 2A, we found that age-dependent *p16* promoter methylation is markedly accelerated by the *cis*-element within intestinal tissues. Moreover, this *p16* promoter hypermethylation exhibits a clonal expansion pattern (Fig. 2B). Because methylation was measured at the whole-tissue level, this result indicates that de novo methylation accumulates in long-lived intestinal stem cells. We hypothesized that the clonal expansion of *p16* methylation with age may provide selective advantages for subsequent accumulation of molecular changes to promote colon cancer development.

To then test the functional requirement for *p16* epimutation in intestinal tumorigenesis, we bred the *p16*^*cis*^ allele into *Apc*^*Min/*+^ mice to generate the following groups of animals: (1) *Apc*^*Min/*+^ (control), (2) *Apc*^*Min/*+^; *p16*^*cis/*+^ (*p16* epimutation at one allele), and (3) *Apc*^*Min/*+^; *p16*^*cis/cis*^ (*p16* epimutation at both alleles). As shown in Fig. 2C, we found that *Apc*^*Min/*+^; *p16*^*cis/cis*^ mice display significantly shortened overall survival compared to *Apc*^*Min/*+^ mice (median 18 *vs*. 25 wk; *P* = 0.001). To further determine the cause of this early mortality in *Apc*^*Min/*+^; *p16*^*cis/cis*^ mice, we assessed the whole bowel tumor formation under a dissecting microscope (Fig. S1A). Notably, we detected a greater number of tumors within the distal regions of the small intestine in *Apc*^*Min/*+^; *p16*^*cis/cis*^ mice than in both *Apc*^*Min/*+^ and *Apc*^*Min/*+^; *p16*^*cis/*+^ mice at 15 wk of age (Fig. 2D). Moreover, tumors in *Apc*^*Min/*+^; *p16*^*cis/cis*^ mice were found to be significantly larger than those in *Apc*^*Min/*+^ and *Apc*^*Min/*+^; *p16*^*cis/*+^ mice (Fig. 2E). Interestingly, we also observed a twofold increase in tumor number within the colons of *Apc*^*Min/*+^; *p16*^*cis/cis*^ mice relative to the other two groups (Fig. 2F). Histological analysis further revealed a substantially increased incidence of high-grade dysplasia and intramucosal carcinoma in colons from *Apc*^*Min/*+^; *p16*^*cis/cis*^ mice relative to *Apc*^*Min/*+^ mice (71% *vs*. 33%). In addition, *Apc*^*Min/*+^; *p16*^*cis/cis*^ mice display aggressive pathological features, such as focal invasion of muscularis mucosa, a hallmark of malignancy that is not present in *Apc*^*Min/*+^ mice (Fig. 2G, Fig. S1B-C). Collectively, these observations from our in vivo model system demonstrate a direct pathogenic role for age-dependent *p16* epimutation in CRC development.

### *p16* epimutation in *Apc*-mutant colon tumors induces proinflammatory immune responses

**Fig. 3:**
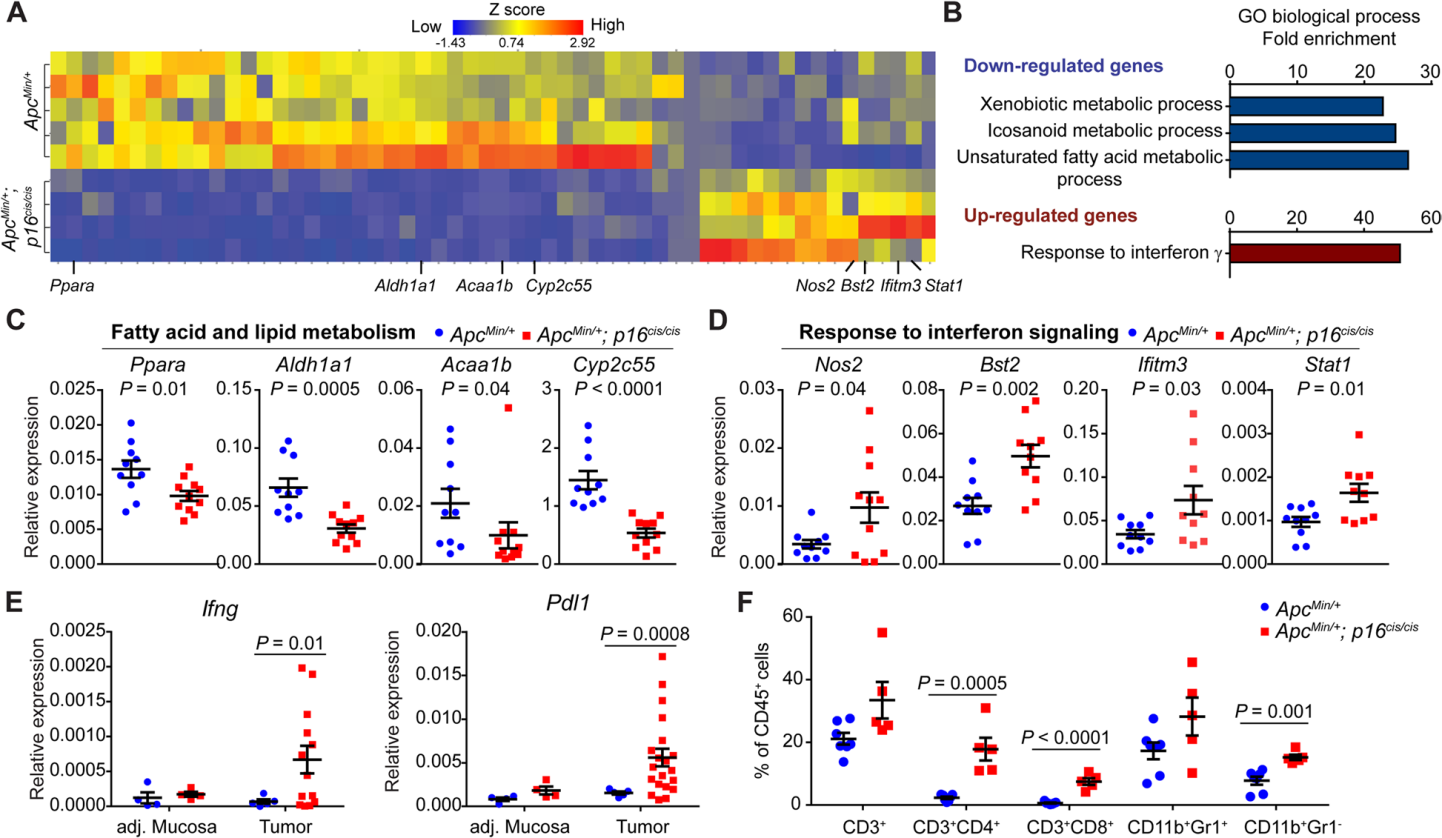
*p16* epimutation promotes inflammatory immune responses in *Apc* mutant colon tumors. **A** Heatmap of DEGs identified by RNA-seq in the normal-appearing colonic mucosa of *Apc*^*Min/*+^ ; *p16*^*cis/cis*^ mice compared with *Apc*^*Min/*+^ mice. We used DESeq2 for DEG analysis with a fold change of > 2 and multiple-testing adjusted *P* value < 0.05. **B** DAVID functional GO analysis of down- and up-regulated DEGs. Significantly enriched terms (FDR < 0.05) are shown. **C** Down-regulation of genes involved in fatty acid and lipid metabolism was confirmed by qRT-PCR. **D** Up-regulation of IFN-γ-stimulated genes in colonic mucosa from *Apc*^*Min/*+^ ; *p16*^*cis/cis*^ compared to *Apc*^*Min/*+^ mice. **E** Up-regulation of *Ifng* and *Pdl1* in mouse colon tumors with *p16* epimutation. **F** Flow cytometric analysis of tumor-infiltrating immune cells. CD4^+^ and CD8^+^ T cells, as well as monocytes (CD11b + Gr1-), increased in *Apc*^*Min/*+^ ; *p16*^*cis/cis*^ colon tumors compared to those from *Apc*^*Min/*+^ mice. For figures (**C**–**F**), data are shown as mean ± SEM with individual values. *P* values were determined by a two-tailed Student’s *t*-test

To identify genes regulated by *p16* epimutation that contribute to intestinal tumorigenesis, we performed RNA sequencing analysis (RNA-seq) and compared transcriptome profiles of colonic mucosa from *Apc*^*Min/*+^; *p16*^*cis/cis*^ mice with those from *Apc*^*Min/*+^ mice at 15 wk of age. From this analysis, we identified a total of 103 differentially expressed genes (DEGs), including 77 downregulated and 26 up-regulated genes (Fig. 3A). Gene Ontology (GO) analysis of downregulated DEGs revealed enrichment of terms associated with cellular metabolic processes, particularly those involved in fatty acid and lipid metabolism (Fig. 3B). We then validated our RNA-seq data by quantitative reverse transcription (qRT)-PCR and confirmed that the genes involved in fatty acid oxidation (*i.e., Ppara*, *Aldh1a1*, *Acaa1b*, and *Cyp2c55*) have significantly reduced expression in *Apc*^*Min/*+^; *p16*^*cis/cis*^ compared to *Apc*^*Min/*+^ mice (Fig. 3C). Interestingly, for the up-regulated DEGs, we detected striking enrichment (50-fold increase) in IFN-γ responsive genes (Fig. 3B). Indeed, qRT-PCR confirmed that expression levels of IFN-γ-stimulated genes, including *Nos2*, *Bst2*, *Ifitm3*, and *Stat1*, are significantly increased in colonic mucosa from *Apc*^*Min/*+^; *p16*^*cis/cis*^ relative to *Apc*^*Min/*+^ mice (Fig. 3D).

Sustained IFN-γ signaling is known to upregulate expression of the immune checkpoint protein PD-L1, which suppresses T cell responses through binding to its ligand, programmed cell death protein 1 (PD-1). We therefore measured the expression levels of *Ifng*, *Pdl1*, and *Pd1* in both colon tumors and adjacent normal mucosa from *Apc*^*Min/*+^ and *Apc*^*Min/*+^; *p16*^*cis/cis*^ mice. Consistent with our RNA-seq data, both *Ifng* and *Pdl1* were found to be specifically up-regulated in colon tumors from *Apc*^*Min/*+^; *p16*^*cis/cis*^ mice (Fig. 3E). In contrast, no appreciable difference in *Pd1* expression between the two groups was detected. To evaluate the clinical significance of these observations, we analyzed human CRC data from The Cancer Genome Atlas (TCGA) and found a weak but significant positive correlation between *p16* epimutation and *PDL1* mRNA expression (*n* = 633, R = 0.21, *P* = 0.00004 by Spearman’s rank correlation test) (Fig. S2A). Interestingly, the correlation becomes more prominent in *KRAS* WT CRCs (*n* = 329, R = 0.31, *P* = 0.000005 by Spearman’s rank correlation test) (Fig. S2B), as well as in a subset of *KRAS* WT CRCs with *APC* mutation (*n* = 161, R = 0.23, *P* = 0.003 by Spearman’s rank correlation test) (Fig. S2C).

We next compared the immune cell profiles in *Apc*^*Min/*+^ and *Apc*^*Min/*+^; *p16*^*cis/cis*^ colon tumors using flow cytometry. Previous studies have reported that although *Apc*^*Min/*+^ mice have defects in hematopoietic stem cells, they show no intestinal inflammation or major alterations in intestinal immune function relative to WT mice [45, 46]. Consistent with the previous report [46], we detected similarly low frequencies of CD3^+^CD4^+^ and CD3^+^CD8^+^ T cells in *Apc*^*Min/*+^ colon tumors (average of 2.3% and 0.6%, respectively). Conversely, we found that both CD3^+^CD4^+^ and CD3^+^CD8^+^ T cells are significantly increased in *Apc*^*Min/*+^; *p16*^*cis/cis*^ colon tumors (average of 17.8% and 7.5%, respectively) (Fig. 3F). In addition, frequencies of monocyte-derived myeloid-lineage cells (CD11b^+^Gr1^+^ and CD11b^+^Gr1^−^) were found to be increased approximately twofold in *Apc*^*Min/*+^; *p16*^*cis/cis*^ colon tumors. Furthermore, we tested for MSI by PCR in colon tumors from *Apc*^*Min/*+^ and *Apc*^*Min/*+^*; p16*^*cis/cis*^ mice. Using a set of five repeat markers, we found that all the tumors analyzed (*n* = 6) were microsatellite stable (Fig. S3). Thus, the immune phenotype in our mouse colon cancer model is independent of MSI. Together, these observations indicate that *p16* epimutation may modulate TME by activating immune-related pathways to promote malignancy of *Apc*-mutant colon cancer.

### scRNA-seq reveals immunosuppressive T cell subtypes during tumor development

**Fig. 4:**
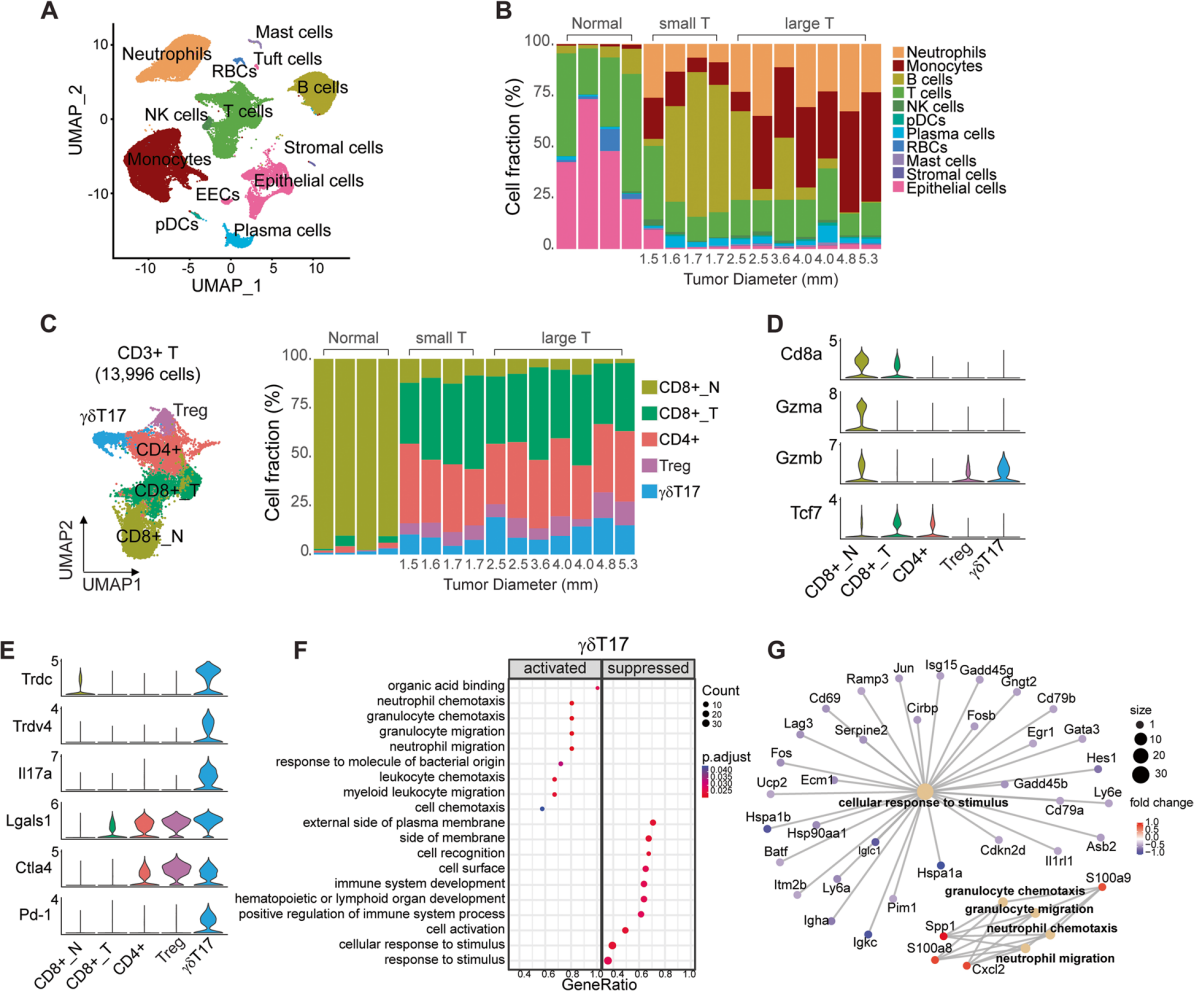
scRNA-seq reveals distinct immunosuppressive T-cell subtypes in *Apc*^*Min/*+^; *p16*^*cis/cis*^ colon tumors during development and progression. **A** UMAP plot showing major cell populations identified from tumors and adjacent normal colon mucosa by canonical cell markers. **B** A bar plot of the proportion of each cell type in individual tumor tissues and adjacent normal colon mucosa. Individual tumors are ordered by diameter and binned into small or large tumors (T). **C** Sub-clustering analysis of CD3^+^ T cells. UMAP plot showing transcriptionally distinct T-cell, subtypes identified in both normal and tumor tissues. For individual normal and tumor samples, the cellular composition of T-cell subtypes is shown by a bar plot. **D** Violin plots showing expression levels of marker genes identifying CD8^+^ cells in each sub-cluster. Compared to the normal *CD8*^+^ T cells (CD8 + _N), tumor-associated *CD8*^+^ T cells (CD8 + _T) show decreased expression of the cytotoxic genes (*Gzma* and *Gzmb*) and increased expression of the exhaustion marker gene (*Tcf7*), indicating a dysfunctional state. **E** Violin plots showing expression levels of the marker genes for γδT17 cells. **F** GO analysis using ClusterProfiler of DEGs in γδT17 cells for early- *vs*. late-stage tumors for DEGs with a log-fold change ≥ 0.25. Correction for multiple testing in the Wilcoxon Rank Sum test and MAST was performed with the Benjamini–Hochberg method. **G** Category net (CNET) plot showing the top pathways and associated DEGs in γδT17 cells during tumor progression. The color of the dots represents the fold change in gene expression, and the size of the dots is proportional to the number of genes enriched with the GO term

Therefore, to comprehensively characterize immune function within the TME in these animals, we performed scRNA-seq on *Apc*^*Min/*+^; *p16*^*cis/cis*^ colon tumors. Because tumor size is associated with malignant histology and nuclear grade, we divided the tumors into two groups: small, early-stage lesions (median diameter = 1.7 mm, *n* = 4) and larger, late-stage larger tumors (median diameter = 4.0 mm, *n* = 7). As a control, we also analyzed cells from adjacent normal colonic mucosa (*n* = 4). After quality filtering and doublet removal, a total of 85,347 cells were analyzed. Based on expression of canonical markers, we identified 11 transcriptionally distinct cell types, including epithelial cells, T cells, B cells, NK cells, neutrophils, monocytes, plasmacytoid (p)DCs, plasma cells, red blood cells, mast cells, and stromal cells (Fig. 4A and Fig. S4). As expected, we found that the expression of *Ifng* is largely restricted to T cells, whereas *Pdl1* is expressed in multiple cell types, including neutrophils and monocytes (Fig. S5). We also detected almost all immune cell types within each individual tumor lesion; however, these immune cell clusters were found to be present in significantly different proportions in early- *vs*. late-stage tumor lesions (Fig. 4B). B cells are the most common cell type in early-stage tumors (56% of 27,945 cells analyzed) but markedly decreased in late-stage tumors (14% of 47,687 cells analyzed). In addition, we detected a rapid expansion of monocytes in late-stage tumors (30% in late-stage tumors *vs*. 10% in early-stage tumors). In contrast, T- cell abundance was found to be relatively unchanged during tumor progression (15% in early-stage tumors and 18% in late-stage tumors).

We then explored T cell status during tumor initiation and progression. Sub-clustering of 13,996 *CD3*^+^ T cells identified five major clusters, which we annotated based on canonical T cell markers. Among them, we detected two clusters of *CD8*^+^ T cells: one preferentially present in adjacent normal tissues and another enriched in tumors (Fig. 4C and Fig. S6A). We found that genes associated with cytotoxic T cell functions (*e.g., Gzma* and *Gzmb*) are highly expressed in the normal *CD8*^+^ T cells. In contrast, tumor-associated *CD8*^+^ T cells show substantially decreased expression of cytotoxic genes and higher expression of the exhaustion marker gene *Tcf7*, indicating a dysfunctional state (Fig. 4D). Comparative transcriptome analysis of *CD8*^+^ T cells from late- *vs*. early-stage tumors further revealed exhaustion-driving transcriptional pathways in cells from late-stage tumors, including upregulation of inflammatory response and downregulation of ribosome biogenesis genes (*e.g*., ribosome assembly and ribonucleoprotein complex translation) (Fig. S6B). We also identified two clusters of tumor-specific *CD4*^+^ T cells, including a population of Tregs expressing the Treg-defining transcriptional factor *Foxp3* (Fig. S7A). These *Foxp3*^+^ Tregs also exhibit high expression of interleukin 2 receptor α-chain (*Il2ra*), members of TNF receptor superfamily (*Tnfrsf4* and *Tnfrsf9*), and genes involved in immune suppression (*Lgals1*, *Areg*, and *Ctla4*) (Fig. 4E and Fig. S7A). Moreover, GO analysis of DEGs from late- *vs*. early-stage *Foxp3*^+^ Tregs revealed downregulation of genes involved in adaptive immune response and regulation of leukocyte activation, which is consistent with the immunosuppressive functions of Tregs (Fig. S7B-C). Similarly, the *CD4*^+^ T cells showed reduced immune responses during tumor progression (Fig. S7D-E).

In addition, we identified a unique tumor-enriched population of *CD4*^−^*CD8*^−^ T cells, exhibiting strong expression of T cell receptor γδ genes (*Trdc* and *Trdv4*) and the pro-inflammatory cytokine *Il17*; these were therefore annotated as γδT17 cells (Fig. 4E). Recent studies have reported that γδT17 cells can promote tumor growth by functioning as Treg-like cells [47, 48]. Indeed, we found that γδT17 cells in tumor tissue are characterized by elevated expression of Treg signature genes, such as *Lgals1*and *Ctla4* (Fig. 4E). Notably, the immune checkpoint molecule *Pd1* was also found to be highly expressed in γδT17 cells (Fig. 4E). Differential gene expression analysis further revealed that γδT17 cells possess both inflammatory and regulatory properties during tumor progression (Fig. 4F), with significant upregulation of genes related to chemotaxis (*e.g.*, neutrophil migration and leukocyte chemotaxis) and downregulation of immune responses (*e.g*., cell recognition, cell activation, and cellular response to stimulus) (Fig. 4G). Taken together, these data show that, in the context of inflammation, dysfunctional *CD8*^+^ T cells, together with *Foxp3*^+^ Tregs and γδT17 cells, contribute to the immunosuppressive TME in colon tumors from *Apc*^*Min/*+^; *p16*^*cis/cis*^ mice.

### Combination therapy improves survival in mice with *Apc* mutation and *p16* epimutation

**Fig. 5:**
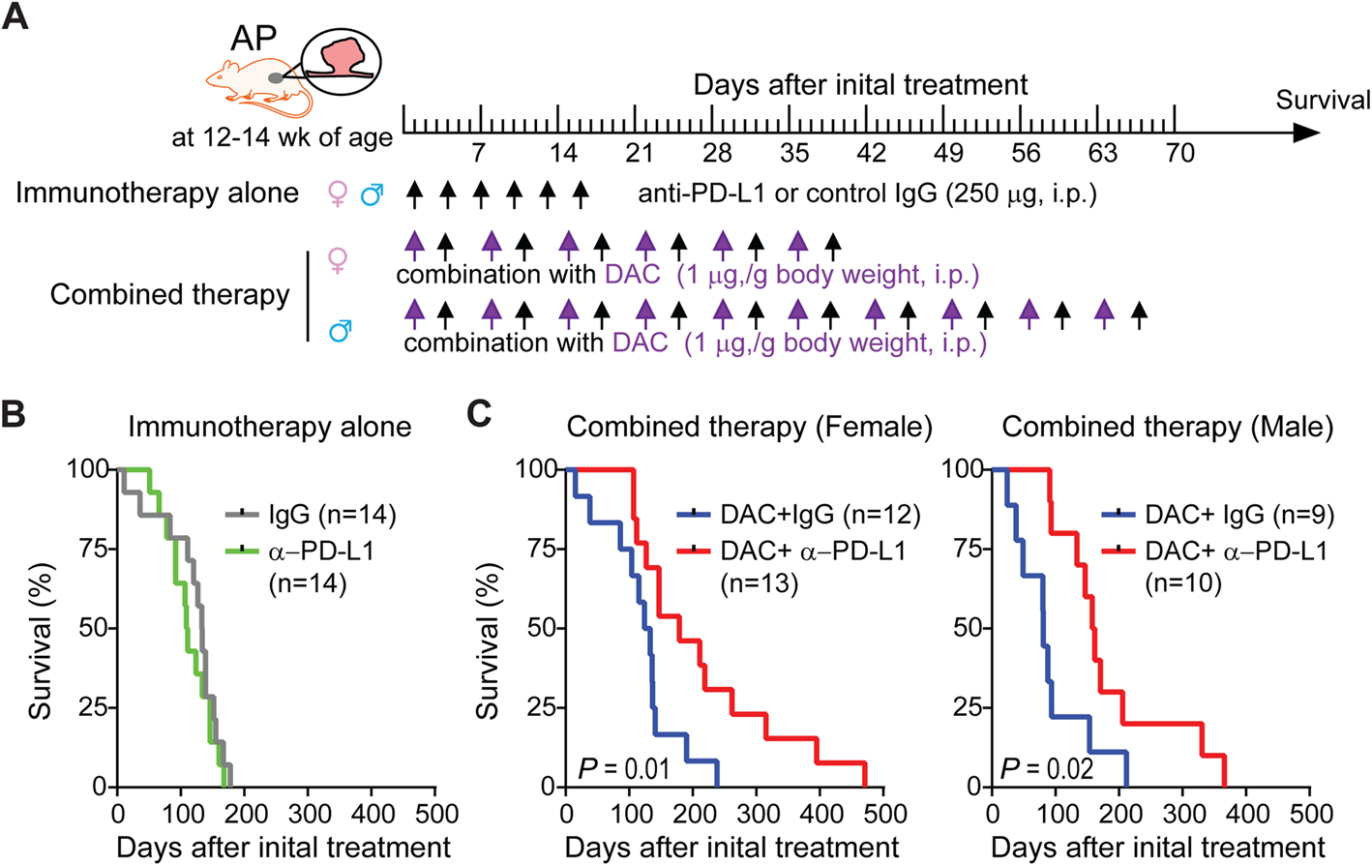
A combined epigenetic and immune therapy improves survival in *Apc*^*Min/*+^; *p16*^*cis/cis*^ mice. **A** Study design and treatment scheme for single anti-PD-L1 mAB therapy *vs.* a combination of the epigenetic hypomethylating agent DAC and anti-PD-L1 mAB. **B** Kaplan Meier survival analysis after anti-PD-L1 or control IgG treatments in *Apc*^*Min/*+^; *p16*^*cis/cis*^ mice. **C** Kaplan Meier survival analysis of *Apc*^*Min/*+^; *p16*^*cis/cis*^ mice treated with DAC plus control IgG *vs.* DAC plus PD-L1. Survival curves were compared using a log-rank test

Based on the above data showing an immunosuppressive phenotype in our unique mouse model of CRC, we next tested whether administration of single-agent anti-PD-L1 therapy at an early stage of tumor progression can effectively control intestinal tumor growth and improve survival of *Apc*^*Min/*+^; *p16*^*cis/cis*^ mice. To this end, *Apc*^*Min/*+^; *p16*^*cis/cis*^ mice (*n* = 28) at 12–14 wk of age were randomly divided into two groups and treated with 250 μg of either anti-PD-L1 or IgG2b isotype control. Treatments were given by intraperitoneal (i.p.) injection every 3 days for a total of six treatments (Fig. 5A). We found that both the drug dosage and frequency of administration were well tolerated in our mouse models. However, we observed no statistically significant difference in median survival between the two treatment groups (110 days *vs*. 133 days,* P* = 0.45 by the log–rank test; Fig. 5B). In addition, we found that PD-L1 blockade has no effect on tumor number or size, with profiles essentially identical to those in control-IgG treated mice.

We then tested whether combined epigenetic and immunotherapy is more efficacious than PD-L1 blockade alone. For these experiments, *Apc*^*Min/*+^; *p16*^*cis/cis*^ mice (*n* = 44) were treated with DAC (1.0 mg/kg, i.p.) on Monday, followed by either anti-PD-L1 or control IgG on Thursday. This dosing schedule was based on the published works indicating that it is well tolerated in mice and rats [49–51] (*i.e.,* resulting in no weight loss or premature mortality) and within the range being tested in human clinical trials. Because male mice at this age weigh more than female mice, we treated females for 6 consecutive weeks and males for an additional 4 weeks. We observed similar survival curves with DAC plus control IgG treatment compared to PD-L1 treatment alone, for both male and female mice, indicating no survival benefit for single-agent DAC therapy. In contrast, we found that DAC plus anti-PD-L1 treatment significantly prolongs survival in *Apc*^*Min/*+^; *p16*^*cis/cis*^ mice, showing a similar effect in both sexes (Fig. 5C). Furthermore, when mice were euthanized at the end of study, we observed significant decreases in both tumor number and size in mice treated with DAC and anti-PD-L1 antibody (Fig. S8). Together, these results demonstrate that combination therapy is more effective than either DAC or anti-PD-L1 treatment alone in our mouse CRC model of *Apc* mutation and *p16* epimutation.

**Fig. 6:**
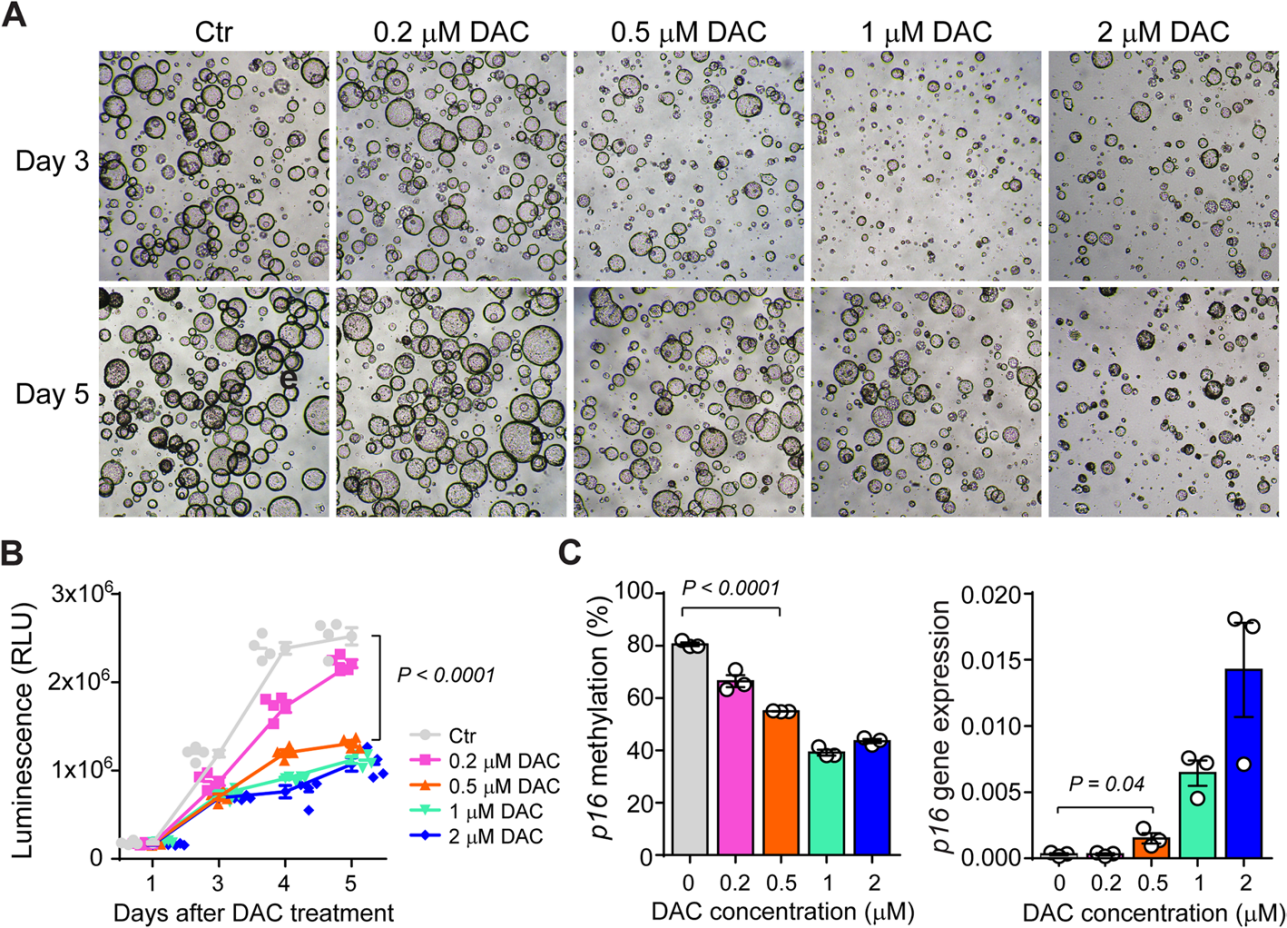
DAC treatment reactivates *p16* and suppresses tumor growth in colon tumor organoids. **A** Images of colon tumor organoids derived from *Apc*^*Min/*+^; *p16*^*cis/cis*^ mice after treatment with DAC at the concentrations shown on days 3 and 5. **B** A dose-dependent decrease in proliferation was observed in tumor organoids treated with DAC. **C** Low-dose DAC (0.5 μM) was sufficient to induce *p16* promoter demethylation and gene reactivation. Error bars represent SEMs of three replicate experiments. Mean values were compared between the 0.5 μM DAC treatment and control (PBS) treatment groups. *P* values were determined by a two-tailed Student’s *t*-test

Recent studies have suggested that the efficacy of PD-L1 blockade requires tumor cell-intrinsic cell-cycle arrest to achieve long-lasting responses [52, 53]. Therefore, based on our data showing that DAC can reactivate epigenetically silenced *p16*, we characterized the direct effects of DAC treatment on tumor cells using an ex vivo organoid system. To this end, we generated colonic tumor organoids from *Apc*^*Min/*+^; *p16*^*cis/cis*^ mice and monitored the percentage of organoids surviving after DAC treatment at days 1, 3, 4, and 5. These time-points were chosen based on established kinetics in intestinal stem cell division and organoid growth [54]. As expected, we observed significantly decreased cell counts in response to DAC treatment, starting at day 3, in a dose-dependent manner (Fig. 6A). Interestingly, we further found that at day 5, DAC at the low dose of 0.5 μM is sufficient to stably inhibit cell proliferation (Fig. 6B). Moreover, consistent with the observed phenotype, we saw significantly decreased *p16* promoter methylation, as well as restoration of *p16* expression (Fig. 6C). On the other hand, DNA hypomethylating drugs can induce global hypomethylation and previous studies suggested that DAC treatment can enhance anti-tumor effects by reactivating transposable elements [26, 55]. To further determine the mechanisms of action for DAC, we additionally measured the expression levels of several murine endogenous retroviruses (ERVs), including *LV30-2*, *MLV*, and *MuRRS*. However, we observed no significant changes in ERV expression levels at the clinically relevant concentration of 0.5 μM (Fig. S9). Furthermore, we analyzed promoter methylation for additional genes that could play a similar tumor-suppressor role in CRC, including the mismatch repair gene *Mlh1*, the WNT signaling regulator *Sfrp1*, and the transcription factor *Gata4*. By comparing the changes of methylation among different promoters, we confirmed that the low dose DAC (0.5 μM) caused the most robust demethylation effect at the *p16* promoter (Fig. S10). Thus, our findings indicate that reversal of epigenetic modification at the *p16* locus suppresses intestinal tumor growth and promotes durable response to immune checkpoint blockade.

## Discussion

The accumulation of epimutations in DNA methylation is a shared molecular phenomenon in both biological aging and cancer development; however, our understanding of how age-related epigenetic changes contribute to tumor evolution and response to therapies is limited. In this study, we have conducted the first functional characterization of age-dependent *p16* epimutation in the adenoma–adenocarcinoma sequence of intestinal tumorigenesis that begins with *Apc* mutation. We chose the *Apc*^*Min/*+^ model because somatic inactivation of the WT *Apc* allele is necessary for tumorigenesis and occurs naturally in mice as they age. This therefore provides an excellent in vivo system to study the biological consequences of accumulated genetic and epigenetic defects, which are often present in human cancers. To elucidate the effect of *p16* epimutation, we applied our previously established mouse model, in which targeted knock-in of the promethylation *cis*-element specifically induces *p16* promoter hypermethylation, particularly in proliferative tissues during aging. Characterization of MEFs derived from this model revealed that induced *p16* epimutation recapitulates key features of epigenetic silencing, resulting in downregulation of *p16* expression. In addition, using pharmacological inhibition of DNA methylation and CRISPR-guided demethylation, we showed that engineered promoter methylation directly controls *p16* gene activity, indicating that this system effectively models *p16* epimutation. Critically, combining the genetically and epigenetically engineered mouse models revealed that *p16* epimutation exacerbates the intestinal tumorigenesis initiated by *Apc* mutation, thus directly linking CRC development and progression to an epigenetic aging marker.

Previous studies [56, 57] have reported that *p16* epimutation functions to control cell cycle progression in human fibroblasts and migration phenotype of cancer cell lines. Here, to investigate how this modification exacerbates intestinal tumorigenesis, we performed RNA-seq analysis of colon tumors from *Apc*-mutant mice with and without *p16* epimutation. Our results showed that *p16* epimutation is associated with increased expression of IFN-γ stimulated genes in colonic mucosa at early stage of tumorigenesis. It is well established that IFN-γ signaling critically regulates mucosal inflammatory processes. Further, although acute IFN-γ signaling is essential for anti-tumor immunity, previous studies have demonstrated that prolonged activation of the IFN-γ signaling pathway can upregulate expression of the immune checkpoint inhibitor PD-L1, leading to tumor immune evasion [58–60]. Indeed, we detected a significant positive correlation between *p16* epimutation and *PDL1* expression in both human and mouse CRCs. Moreover, the observation that *p16* epimutation promotes tumor progression, despite an abundance of T cell infiltration, is consistent with a model in which the IFN-γ/PD-L1 axis can switch immune cell phenotypes from a pro-inflammatory to an immunosuppressive state. Our findings therefore suggest that *p16* epimutation modulates the immune state of the TME.

We further explored this phenomenon using scRNA-seq, which revealed the striking observation that colon tumors with defined mutation (*Apc*) and *p16* epimutation contain a high infiltration of T cell subtypes. Specifically, we detected the presence of *Foxp3*^+^ Tregs and γδT17 cells at the early phase of tumorigenesis. These *Foxp3*^+^ Tregs express high levels of *Il2ra*, T cell costimulatory receptors (*Tnfrsf4* and *Tnfrsf9*), and regulatory molecules, such as *Areg* and *Ctla4*, all of which can contribute to the immunosuppressive activity of these cells. For example, IL-2R signaling is required for both Treg survival and suppression of CD8^+^ effector T cells [61], and CTLA-4 plays a key role in Treg-mediated suppression, in part, by inhibiting the activity of antigen-presenting cells via its interactions with both CD80 and CD86 [62]. Thus, elevated levels of *Foxp3*^+^ Tregs likely promote immunosuppression within the TME. In parallel, γδT17 cells may directly enhance tumor-elicited inflammation and colon cancer progression. In human CRCs, infiltration of γδT17 cells is positively correlated with advanced clinicopathological features, including TNM stage, tumor size, and both lymphatic and vascular invasion [48]. In addition, IL-17, a key cytokine produced by γδT17 cells, has been shown to promote tumor growth via the recruitment of myeloid-derived suppressor cells in the TME [47, 63]. Consistent with these findings, at the single-cell resolution, we found that inflammatory γδT17 cells specifically express *Pd1,* as well as key factors that stimulate granulocyte chemotaxis and promote recruitment of neutrophils and monocytes. Nevertheless, further studies utilizing in vitro functional assays, co-culture experiments, and in vivo selective ablations, are needed to uncover the precise roles of dysfunctional and immunosuppressive T cells in *p16* epimutation-driven CRC progression.

Several epigenetic drugs, including DAC, are already in clinical use for treatment of CRC [64, 65]. In addition, anti-PD-1/PD-L1 therapy has emerged as one of the most promising immunotherapies for CRC patients with microsatellite instability (MSI) [66]. However, combinatorial epigenetic immunotherapy regimens have yielded limited response in advanced CRC patients with microsatellite stable (MSS) tumors [67]. Such approaches are also associated with numerous challenges, including a lack of predictive markers and uncertainty regarding the optimal timing for therapy. Notably, our mouse model of MSS tumors driven by defined mutation and epimutation provides an experimentally tractable system in which therapeutic effects can be studied in the natural tumor microenvironment with native stroma and intact immune system. We found that, despite high levels of *Pdl1* expression and T cell infiltration, intestinal tumors from *Apc*-mutant mice with *p16* epimutation are resistant to anti-PD-L1 treatment alone. Interestingly, however, delivery of DAC followed by anti-PD-L1 significantly improves animal survival. Mechanistically, we postulate that *p16* may contribute to IFN-γ-dependent activation of tumor-intrinsic cell death, which is required for stable arrest of cancer cells that escape immune-mediated cytotoxicity and thus, long-term cancer control. Consistent with this, we found that *p16* epimutation leads to senescence bypass in primary MEFs. Moreover, using colon tumor organoids generated from mice with *p16* epimutation, we further confirmed that DAC treatment induces *p16* promoter demethylation and restores tumor-intrinsic cell-cycle regulation. Interestingly, while we observed significant growth inhibitions in the tumor organoids in response to DAC, there is no survival benefit for DAC treatment alone in mice. It is known that the single-agent DAC therapy in vivo requires repetitive cycles for a long duration [68, 69]. However, to avoid the treatment-related toxicity, we stopped the drug administration after 6–10 cycles, which may explain the low efficacy in solid tumors. Nevertheless, our preclinical studies indicate that the epigenetic therapy works best when combines with the anti-PD-L1 immunotherapy. Although beyond the scope of this study, our established clinically relevant and immunocompetent mouse models will enable future studies to probe new and improved hypomethylating agents, such as GSK3685032 which selectively inhibits DNMT1 with lower toxicity [70].

## Conclusions

In summary, we established a mouse model of CRC that combines *Apc* mutation with *p16* epimutation, and we found that age-dependent *p16* epimutation modulates tumor microenvironment to accelerate malignant transformation. At the molecular level, our mouse model closely resembles human CRC. From the therapeutic standpoint, epimutations are reversible, so our model has proven useful as an in vivo preclinical platform for developing novel therapeutics.

## Supplementary Information

**Additional file 1: Figure S1.** *p16 *epimutation accelerates malignant transformation of *Apc*^*Min*^-mediated adenomatous polyps. **Figure S2.** Positive correlation between *p16* promoter methylation and *PDL1 *expression in the TCGA-CRC dataset.** Figure S3. **Mouse colon tumors with combined *Apc* mutation and *p16* epimutation are microsatellite stable (MSS).** Figure S4. **Dot plot visualization of representative marker genes in each cell type. **Figure S5.** UMAP plots showing abundant *Pdl1* expression in the Cd45^+^ tumor-infiltrating immune cells.** Figure S6.** scRNA-seq analysis of CD8^+^ T cells during tumor development and progression.** Figure S7. **scRNA-seq analysis of CD4^+^ T cells, including Tregs, during tumor development and progression. **Figure S8.** Number of intestinal tumors in mice at the end of study at ~30 wk of age. **Figure S9.** Expression of murine ERVs in colon tumor organoids after DAC treatment at day 5. **Figure S10.** The impact of DAC treatment on other CRC-related tumor suppressor genes.

**Additional file 2: Supplementary Table 1.** Primers and PCR conditions for genotyping assays. **Supplementary Table 2.** Sequences of gRNAs for CRISPR-mediated targeted demethylation. **Supplementary Table 3.** Bisulfite-pyrosequencing PCR primers and sequenced regions for quantitative DNA methylation analysis. **Supplementary Table 4.** TaqMan assays and SYBR green primers for qRT-PCR. **Supplementary Table 5. **Microsatellite markers for detection of MSI in mouse tumors.

## Data Availability

The datasets generated and/or analyzed during the current study are available in the GEO database (GSE214032, GSE213568 and GSE213568).

## Abbreviations

CRC: Colorectal cancer
CDKN2A: Cyclin-dependent kinase inhibitor 2A
CGIs: CpG islands
MEF: Mouse embryonic fibroblast
DAC: 5-Aza-2’-deoxycytidine
PD-L1: Programmed death-ligand 1
ERV: Murine endogenous retroviruses
TME: Tumor microenvironment
TNM: The tumor, node, metastasis
MSI: Microsatellite instability
MSS: Microsatellite stable
WGBS: Whole genome bisulfite sequencing
RNA-seq: RNA sequencing
scRNA-seq: Single-cell RNA sequencing
UMAP: Uniform Manifold Approximation and Projection
DEG: Differentially expressed gene
GO: Gene Ontology
KEGG: Kyoto Encyclopedia of Genes and Genomes
GEMS: Gel Bead-in-Emulsions
SEM: Standard error of the mean
