# Supplementary material for: Functional characterization of age-dependent p16 epimutation reveals biological drivers and therapeutic targets for colorectal cancer
Source: J Exp Clin Cancer Res. 2023 May 4;42:113. doi: 10.1186/s13046-023-02689-y (PMC10157929; doi:10.1186/s13046-023-02689-y)
Supplement: Supplementary file 1 — Additional file 1: Figure S1. p16 epimutation accelerates malignant transformation of ApcMin-mediated adenomatous polyps. Figure S2. Positive correlation between p16 promoter methylation and PDL1 expression in the TCGA-CRC dataset. Figure S3. Mouse colon tumors with combined Apc mutation and p16 epimutation are microsatellite stable (MSS). Figure S4. Dot plot visualization of representative marker genes in each cell type. Figure S5. UMAP plots showing abundant Pdl1 expression in the Cd45+ tumor-infiltrating immune cells. Figure S6. scRNA-seq analysis of CD8+ T cells during tumor development and progression. Figure S7. scRNA-seq analysis of CD4+ T cells, including Tregs, during tumor development and progression. Figure S8. Number of intestinal tumors in mice at the end of study at ~30 wk of age. Figure S9. Expression of murine ERVs in colon tumor organoids after DAC treatment at day 5. Figure S10. The impact of DAC treatment on other CRC-related tumor suppressor genes. [file 13046_2023_2689_MOESM1_ESM.pdf]

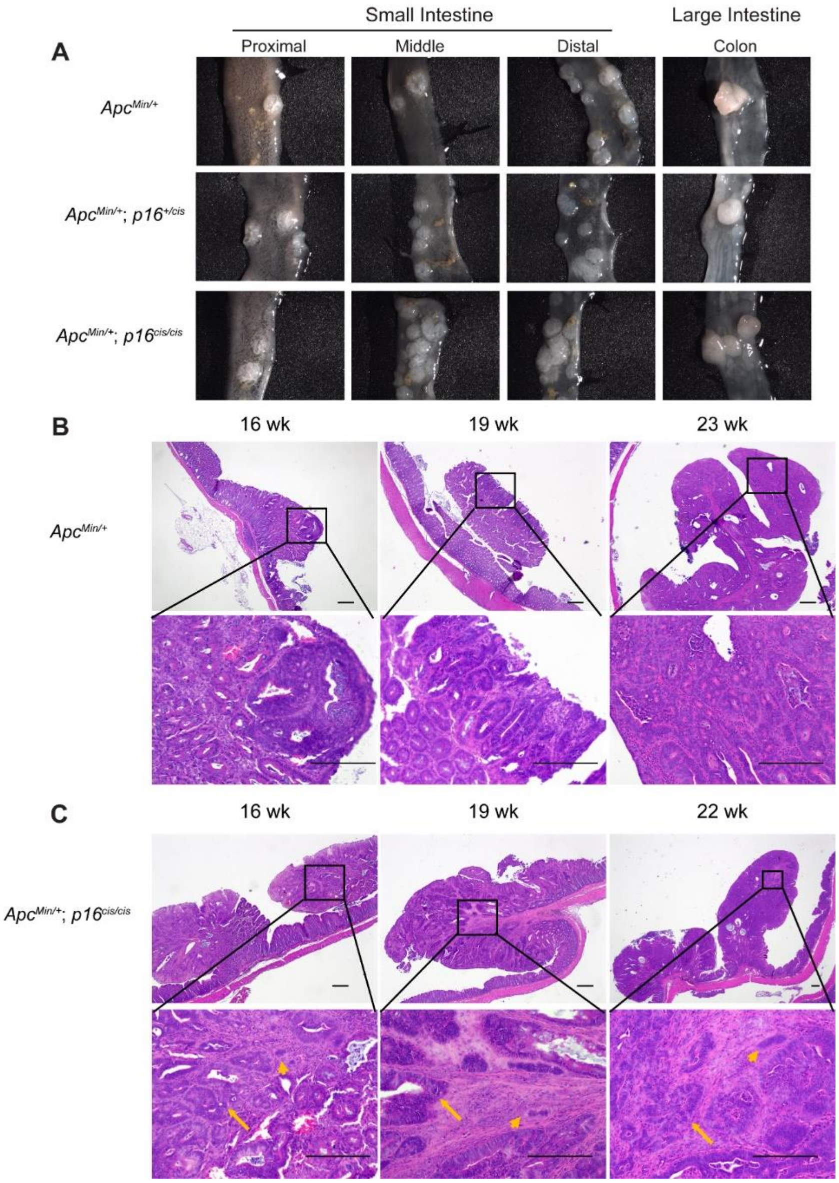

Figure S1. *p16* epimutation accelerates malignant transformation of *Apc*^{Min}-mediated adenomatous polyps. A. Gross examination of the whole bowel tumor formation in *Apc*$^{Min/+}$, *Apc*$^{Min/+}$; *p16*$^{+/cis}$, and *Apc*$^{Min/+}$; *p16*$^{cis/cis}$ mice. Using a dissecting microscope, tumor numbers and sizes were determined within the intestinal track from small intestine to colon. The small intestine was divided into three major segments: proximal, middle, and distal. Representative macroscopic appearance of the tumors is shown. **B.** Time-course analysis of tumor sections from the *Apc*$^{Min/+}$ mice. Three representative colon tumors at the indicated age are shown. H&E staining revealed high-grade dysplasia, characterized by cribriforming glands and focal nuclear pleomorphism. However, histological features of carcinoma were not observed. A high magnification of the boxed area is shown on the bottom. Scale bars: 200 μm. **C.** The presence of carcinoma formation in the *Apc*$^{Min/+}$; *p16*$^{cis/cis}$ mice as early as 16 wk of age. Carcinomas formation in the *Apc*$^{Min/+}$; *p16*$^{cis/cis}$ mouse colons was characterized by angulated, atypical glands (arrow), tumor necrosis, and rare single cells (arrowhead). A high magnification of the boxed area is shown on the bottom. Scale bars: 200 μm.

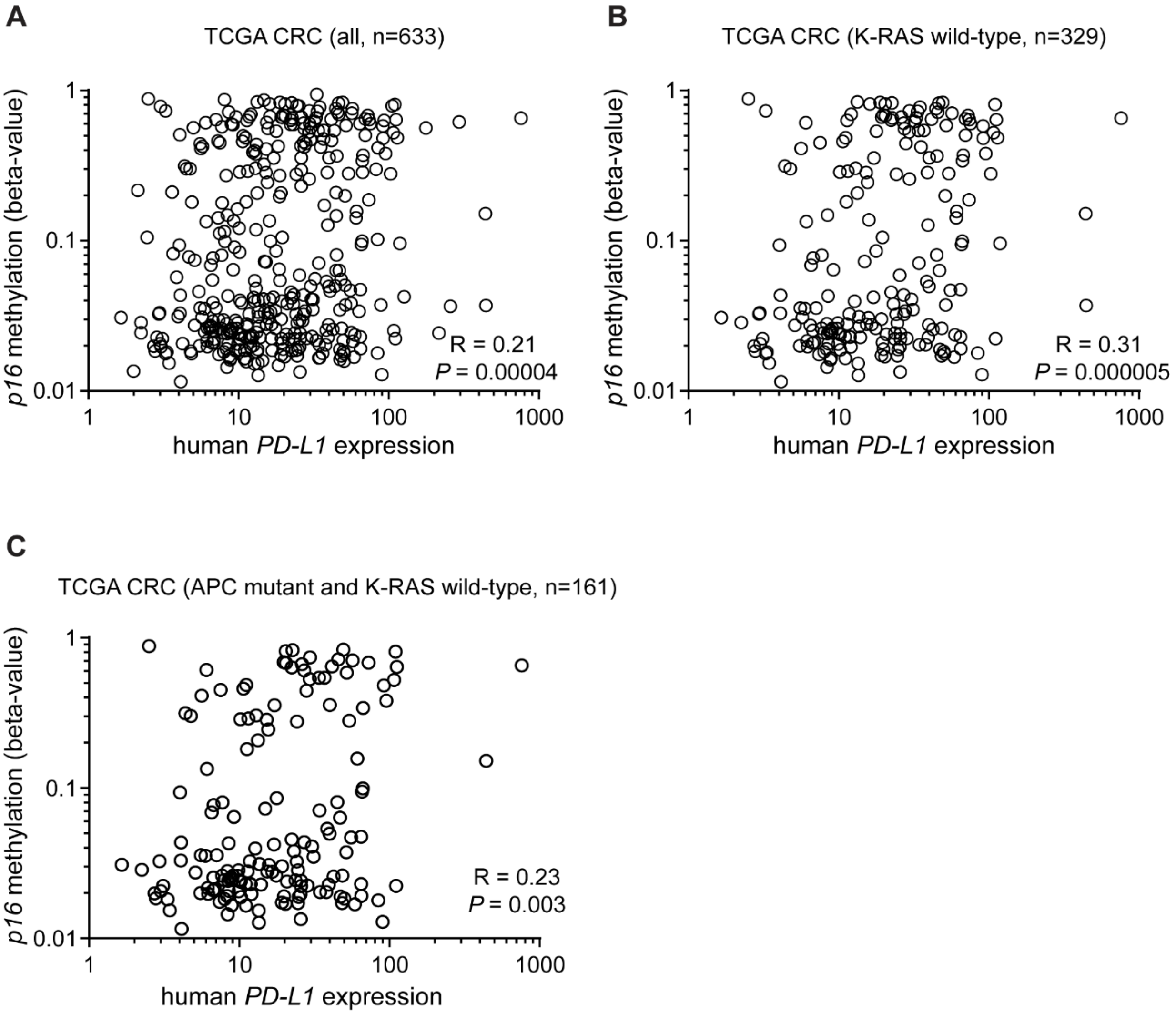

Figure S2. **Positive correlation between *p16* promoter methylation and *PDL1* expression in the TCGA-CRC dataset.** The *p16* methylation was determined by the Illumina methylation probe (cg13601799) located within the promoter CpG island and used routinely to assess *p16* epigenetic silencing (Zhou et al. Nucleic Acids Res. 2018; 46(20):e123). The correlation analysis was based on the TCGA data of all CRC patients **(A),** within the patients with wild-type *K-RAS* **(B),** and in a subset of *K-RAS* wild-type patients with *APC* mutation **(C).**

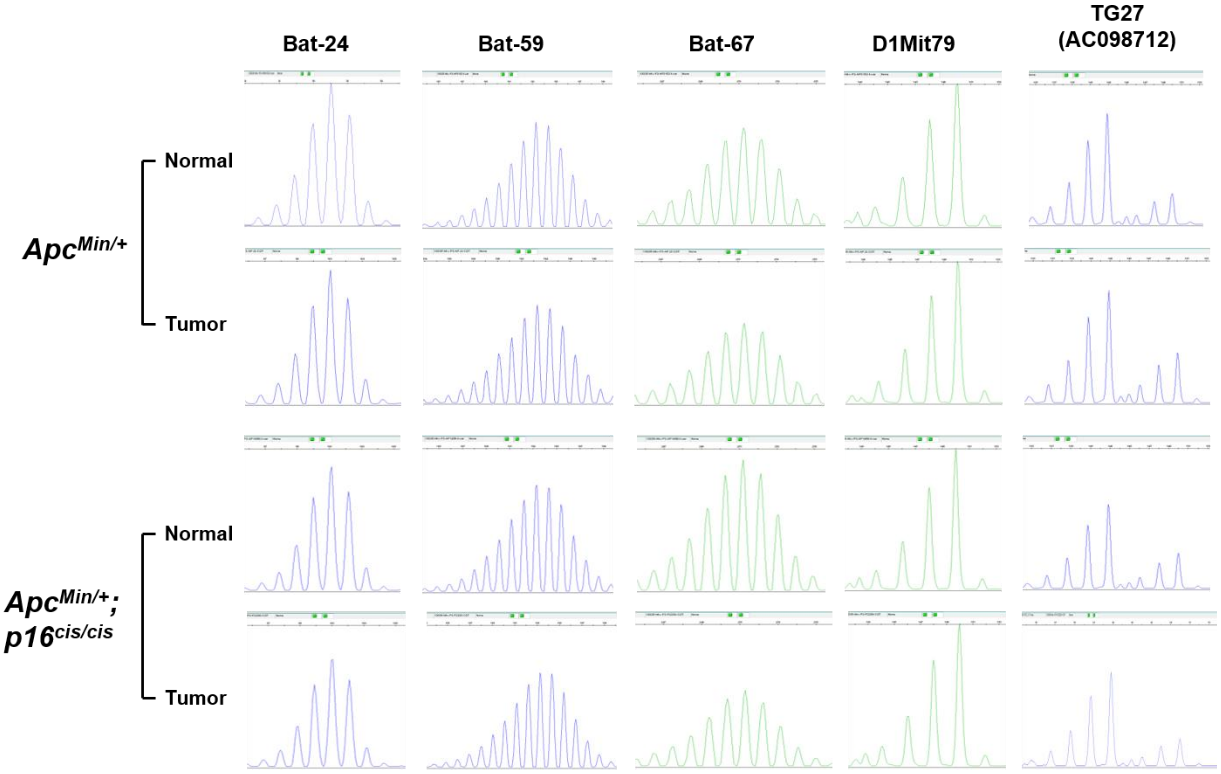

Figure S3. Mouse colon tumors with combined *Apc* mutation and *p16* epimutation are microsatellite stable (MSS). A panel of five microsatellite markers were used for PCR-based MSI analysis. All the tumor samples analyzed (n=6) were classified as MSS, since they showed the same profiles as those seen in the healthy tissues (liver).

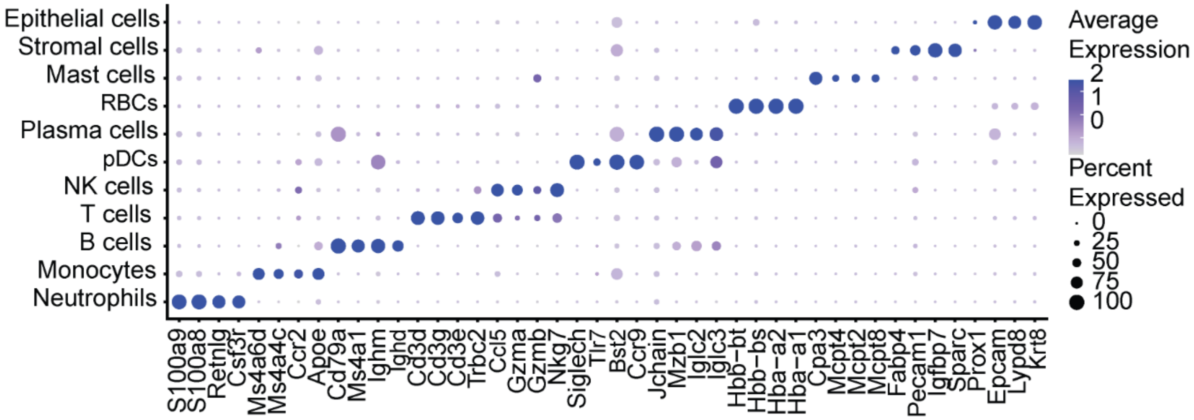

Figure S4. Dot plot visualization of representative marker genes in each cell type.

The color intensity encodes the average expression level, and the size of the dot encodes the percentage of cells within a cell type.

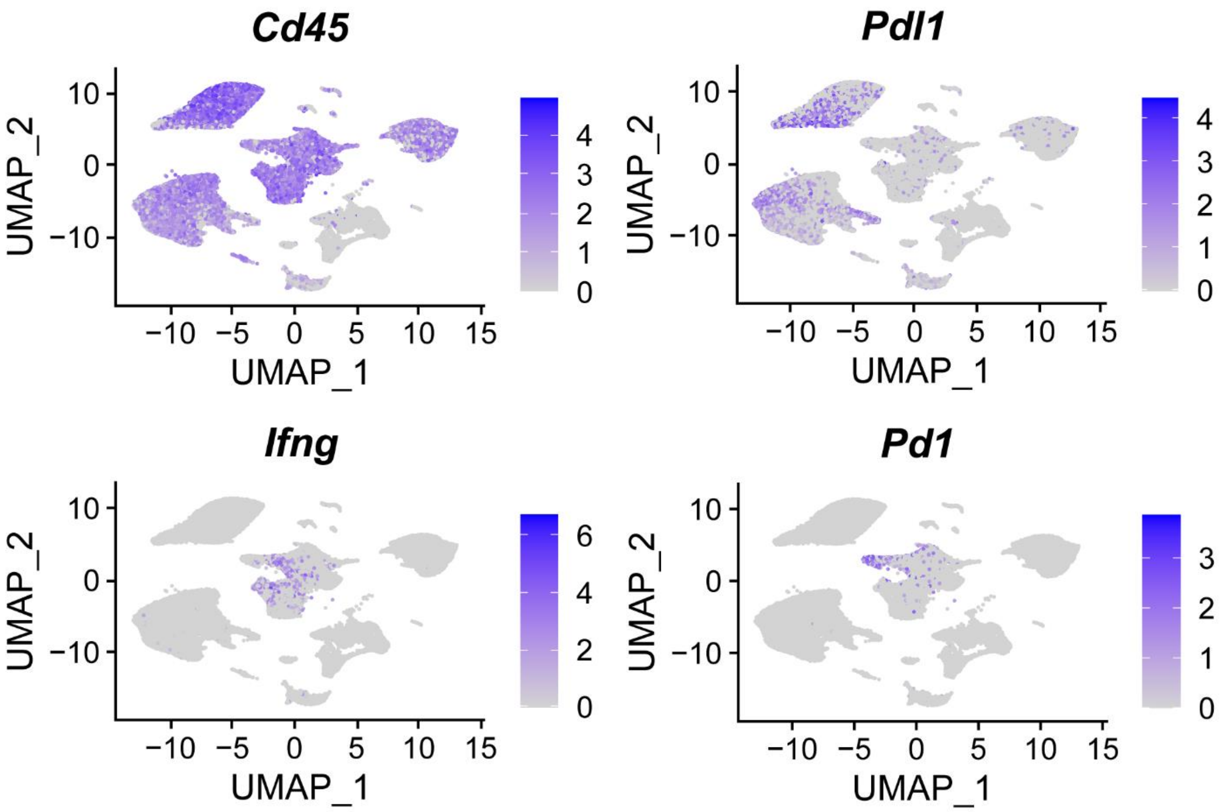

Figure S5. UMAP plots showing abundant *Pdl1* expression in the Cd45^{+} tumor-infiltrating immune cells. UMAP feature plots showing expression patterns for selected genes, including *Cd45*, *Pdl1*, *Ifng*, and *Pd1*. The color intensity indicates the relative expression as indicated by the scale bar.

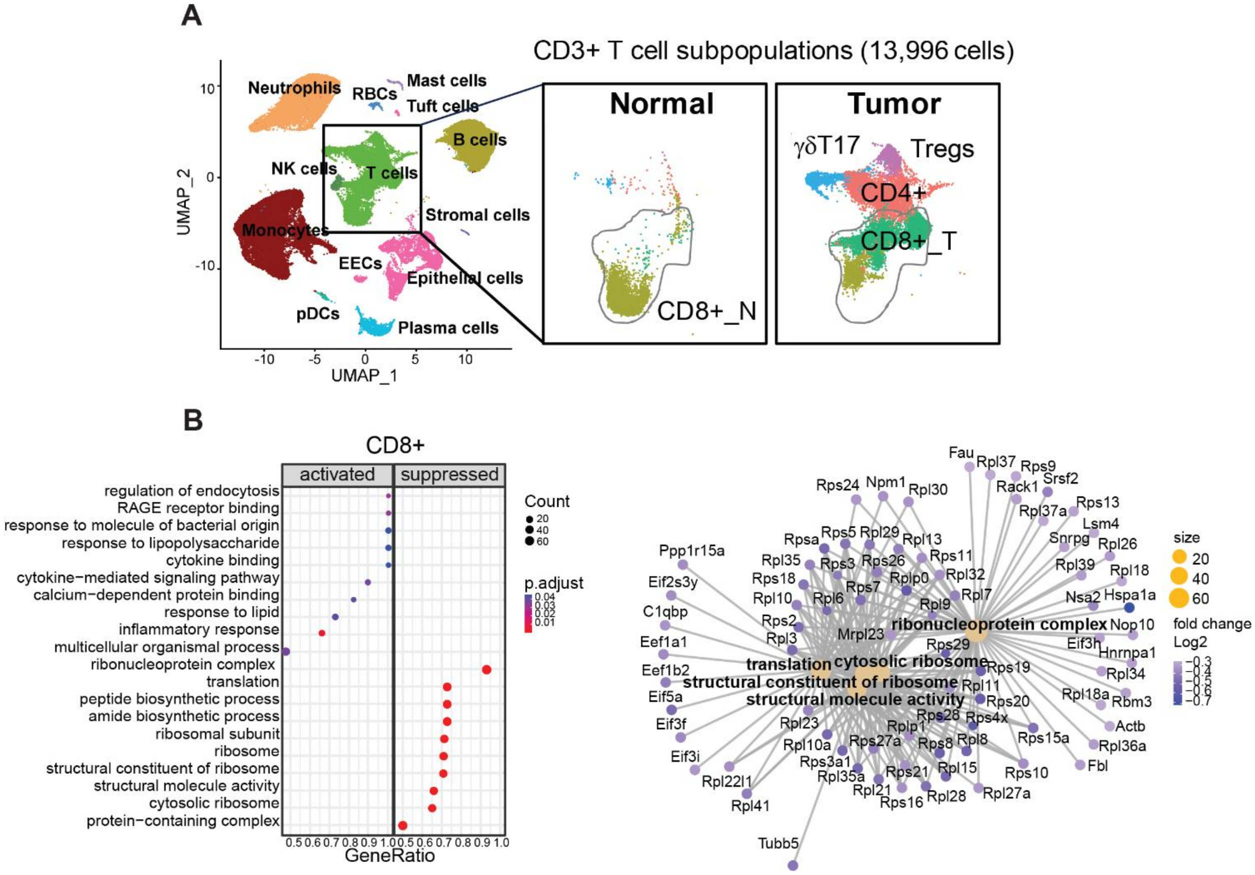

Figure S6. scRNA-seq analysis of CD8$^+$ T cells during tumor development and progression. A. CD3$^+$ T cells were sub-clustered into five major clusters and annotated based on canonical gene markers. UMAP projections of CD3$^+$ T cells according to the tissue of origin indicated that CD8$^+$ T cells identified from adjacent normal mucosa compared to tumor tissues comprise transcriptionally distinct groups. **B.** GO analysis of DEGs in CD8$^+$ cells from early- *vs*. late-stage tumors. The left panel shows the top pathways associated with either activated or suppressed DEGs. The right panel is a CNET plot showing the down-regulation of ribosome biogenesis genes (e.g., ribosome assembly and ribonucleoprotein complex translation).

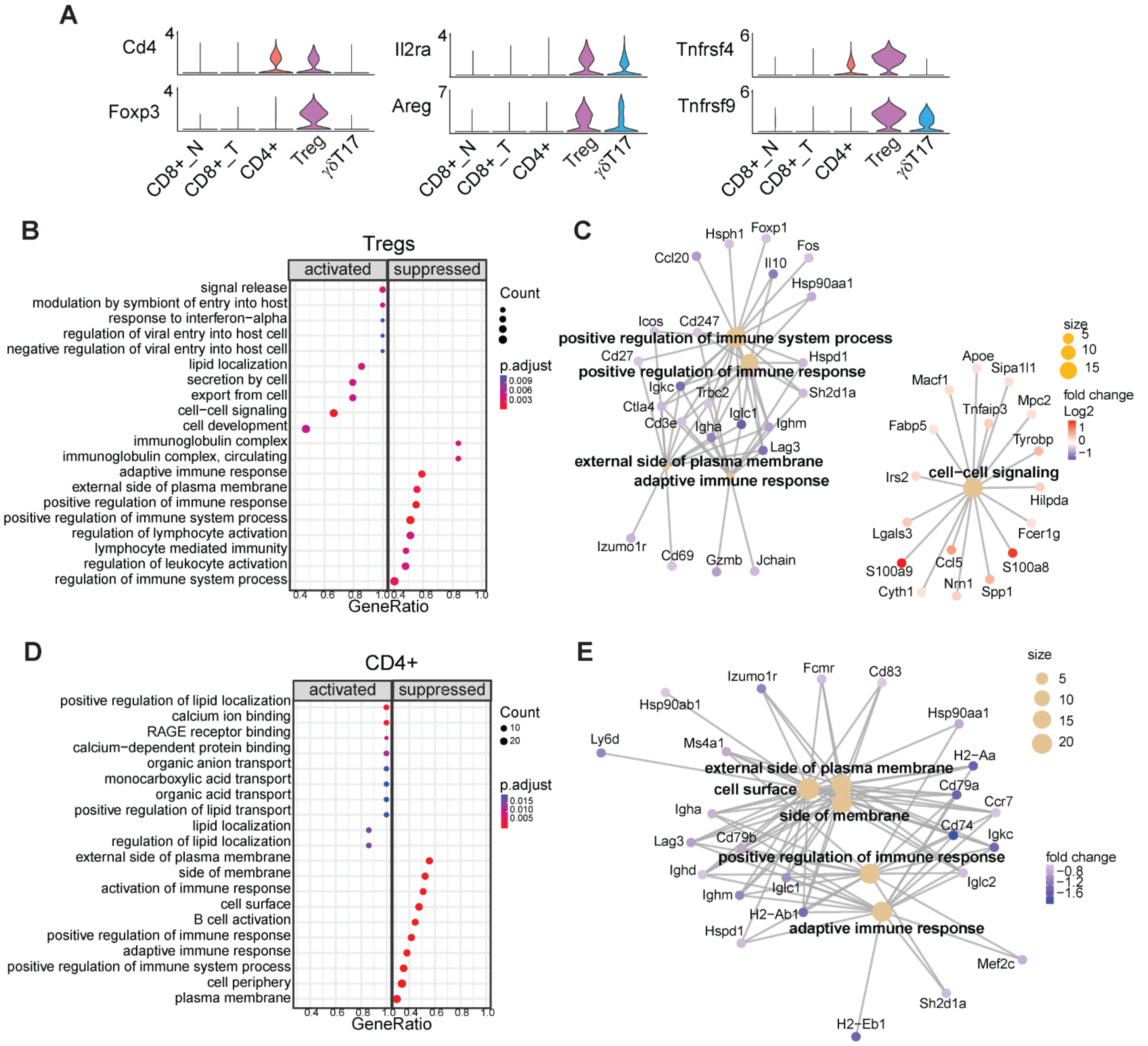

Figure S7. scRNA-seq analysis of CD4⁺ T cells, including Tregs, during tumor development and progression. A. Violin plots showing expression levels of marker genes related to CD4⁺ T cells and Tregs in each sub-cluster. **B.** GO analysis of DEGs in Tregs from early- *vs*. late-stage tumors. **C.** CNET plot showing DEGs in Tregs that were down-regulated in immune responses and up-regulated in cell-cell signaling. **D.** GO analysis of DEGs in CD4⁺ T cells from early- *vs*. late-stage tumors. **E.** Down-regulated DEGs in CD4⁺ T cells were associated with positive regulation of immune response and the adaptive immune response.

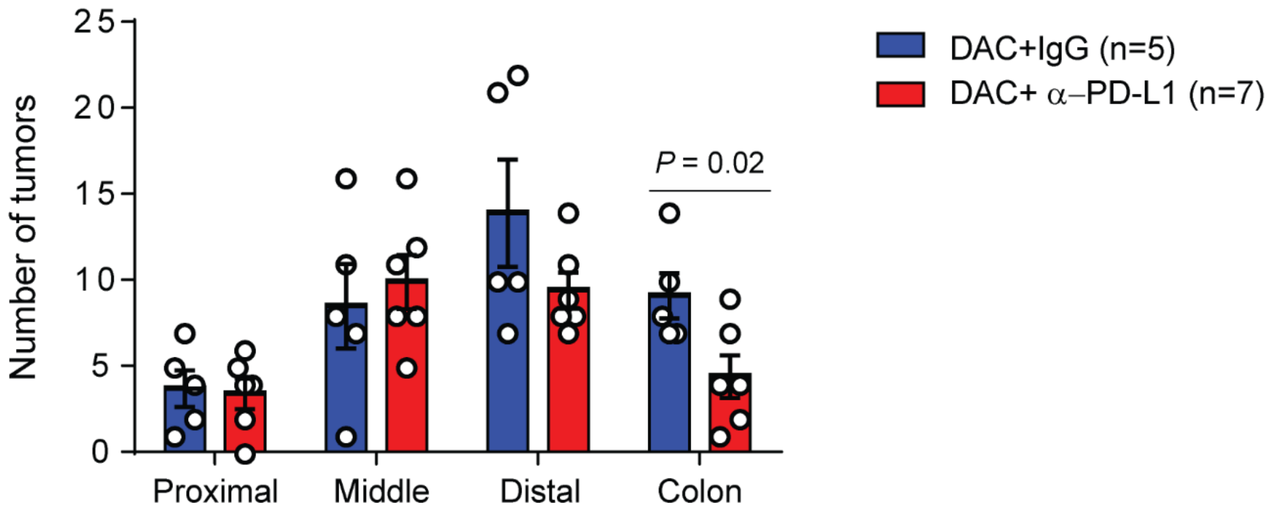

Figure S8. Number of intestinal tumors in mice at the end of study at ~30 wk of age.

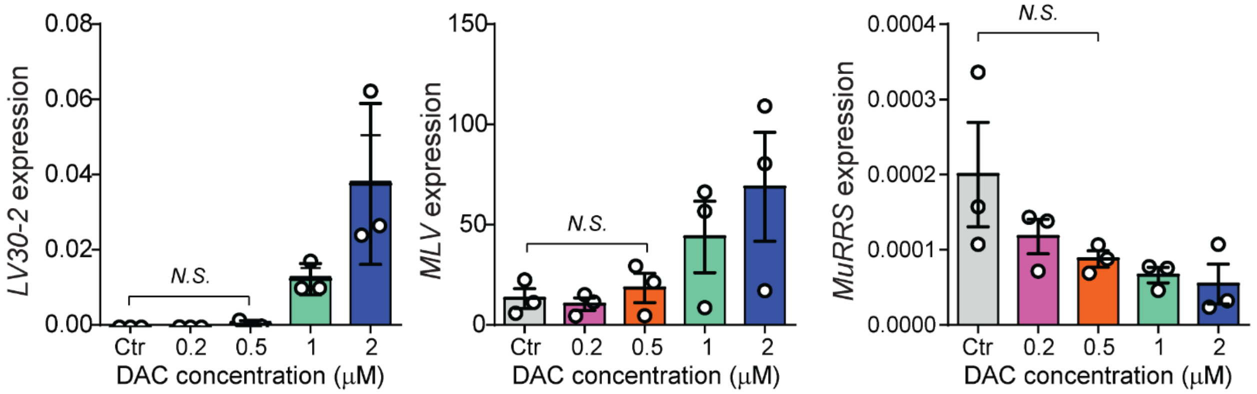

Figure S9. Expression of murine ERVs in colon tumor organoids after DAC treatment at day 5. Relative expression of *LV30-2*, *MLV*, and *MuRRS* was analyzed according to a previous report (Saito et al. Sci Rep., 2016, 6:25311). Expression levels in organoids treated with 0.5 μM DAC were compared with those treated with PBS (Ctr). *P* values were determined by a two-tailed Student's *t*-test. N.S.= not significant.

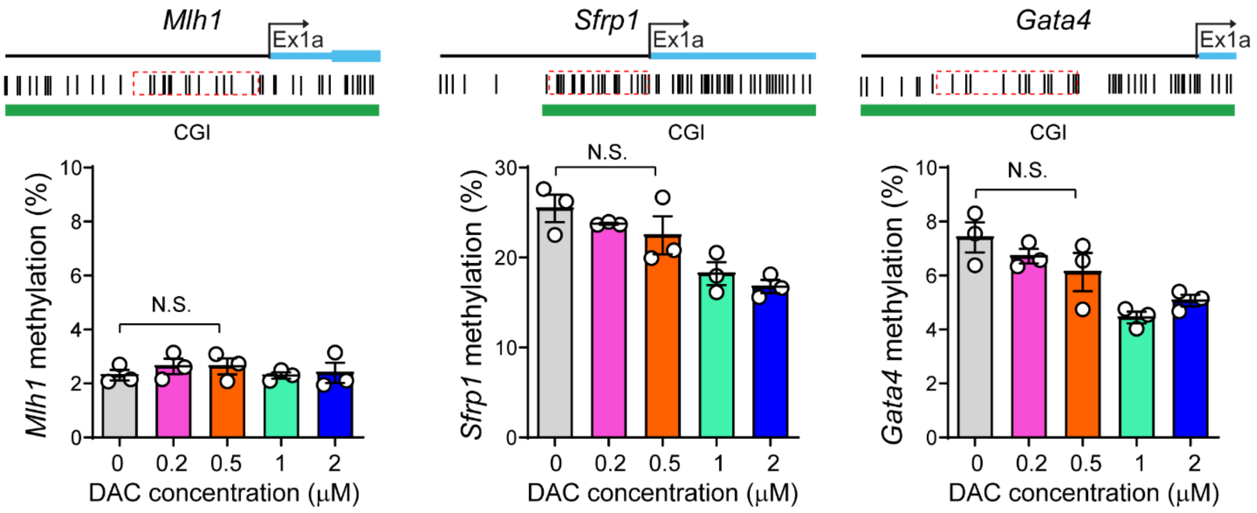

Figure S10. The impact of DAC treatment on other CRC-related tumor suppressor genes. For each gene, a CpG map of the promoter region is shown. Thin blue bars represent the 5'-UTRs and the thick blue box indicate the coding exon (Ex). Each vertical line represents a CpG site and green bars indicate CpG islands (CGIs). Red dotted boxes indicate the regions analyzed for promoter DNA methylation in the *Apc*$^{Min/+}$; *p16*$^{cis/cis}$ colon tumor organoids. At the low dose of 0.5 μM, we did not find significant changes of methylation at the three CRC-related tumor suppressor genes including *Mlh1*, *Sfrp1*, and *Gata4*.
