# Supplementary material for: Functional characterization of age-dependent p16 epimutation reveals biological drivers and therapeutic targets for colorectal cancer
Source: J Exp Clin Cancer Res. 2023 May 4;42:113. doi: 10.1186/s13046-023-02689-y (PMC10157929; doi:10.1186/s13046-023-02689-y)
Supplement: Supplementary file 2 — Additional file 2: Supplementary Table 1. Primers and PCR conditions for genotyping assays. Supplementary Table 2. Sequences of gRNAs for CRISPR-mediated targeted demethylation. Supplementary Table 3. Bisulfite-pyrosequencing PCR primers and sequenced regions for quantitative DNA methylation analysis. Supplementary Table 4. TaqMan assays and SYBR green primers for qRT-PCR. Supplementary Table 5. Microsatellite markers for detection of MSI in mouse tumors. [file 13046_2023_2689_MOESM2_ESM.pdf]

Supplementary Table 1. Primers and PCR conditions for genotyping assays

Primer name	Sequence	Genotype	PCR product (bp)	PCR condition (Tm_cycle)
P16wt-F1	TTTTTAAATCCTCCCTTCTGTCC	WT	156	55°C_35
P16wt-R1	GGGCTGTTGTTTGTTTAAATGAGT			
P16cis-F1	TTTAAATCCTCCCTTCTGTCCA	cis	326	55°C_35
P16cis-R1	AGAGTTACCAGGGATCCACCTAAT			
APC^{min}_mut	TTCTGAGAAAGACAGAAGTTA	WT	No band	55°C_35
APC^{min}_common	TTCCACTTTGGCATAAGGC	Min	320	

Supplementary Table 2. Sequences of gRNAs for CRISPR-mediated targeted demethylation

Target	Sequence
p16-gRNA	CCATTATTGCATTAATCTGA
Ctr-gRNA	GTGCAACGTTTATGCGCAGC

Supplementary Table 3. Bisulfite-pyrosequencing PCR primers and sequenced regions for quantitative DNA methylation analysis (Btn, biotin)

Gene	CpGs (relative to TSS)	PCR		Pyrosequencing	
		PCR primers	Condition (Tm_cycles)	Pyrosequencing primers	Sequence analyzed
p16	-2308 bp to -2264 bp	AGAAGTTATTTAGAATTGAGGTTTTAGAT	55°C_45	AGTATTAGGGATAAATTTT	TYGTTTTTTT GTTTAATTTA ATTTTTTTAG GGTTATTTTG GAATAYGAAT TTTTTTTTGA AATATAGTTA A
		[Btn]ATCCAATATTTCCTCTACTCTATAACC			
p16	-1156 bp to -1010 bp	AGAAAGGGTTATTGTTTTTTTGGT	55°C_45	S1: TTTAAATTTTTTTTTTTGTTTAGTA	TTYGAGATAA TTTYGTATAA TGTATGTTAT AYGAAGTTAT ATYGATTTTT GTTTTGTTAT TTAGGTTG
		[Btn]TTCATCCCAACTACTTAAAAAACT		S2: TTTTTGTATTTTTAGTAGAG	AYGGGTTTTTATTATGTTGGTTAGGT TGAT ATYGTGATTT TTTTGTTTTA GTTTTTTA
p16	-906 bp to -589 bp	AGTATAGGAATTTTATTAGTTAGGTATAT	55°C_45	S1: TTAGGTATATAATATAATTT	YGTATAATGT ATGTTATAYG AAGTTATTAG GTGGATTTTT GGTAATTTTG TTTAAAGYGT GTTTT
				S2: ATTTTTGGTAATTTTGTTTA	AAGYGTGTTT TTTTTTTTGT TTTATAAGAT TGTAAAGAYG TTTTTAAYGA ATAATTTAAA TYGGTGTAAY GTTTATG
		[Btn]AACAACCCTAACTCAAACAAC		S3: AAATGTTTTTGTAAATTTTT	YGTAAAGATT YGGATTTTAT ATTGGGYGTG GTATTTTTTA AAATGAGTTG TT
				S4: AGGAATTTTATTAGTTAGGTATATA	ATATAATTTYGTATAATGTATGTTATAYGA
p16	-814 bp to -589 bp	AGGTGGATTTTTGGTAATTTTGTT	55°C_45	S1: TTTTTGTTTTATAAGATTGTAA	AGAYGTTTTT AAYGAATAAT TTAAATYGGT GTAAYGTTTA TGYGTAGTAT ATTAATTTAT TTAAATAA

		[Btn]AACCCTAACTCAAACAACTCATTTT		S2: TATTTTATAAGTAGATTGTT	TTTYGATGAT TTTATTTYGT TATTTTTTTA TAGTTGTGTA TAG
				S3: AAATGTTTTTGTAAATTTTT	YGTAAAGATT YGGATTTTAT ATTGGGYGTG GTATTTTTTA AAATGAGTTG TT
p16	-433 bp to -313 bp	GTTAGGGTTGTTGGGATTTTAGTT	60°C_45	S1: GTTTTAGTGTAGTGATGAAAATTA	TTTTTTTTYG TTTTTTTAAT ATTTGGGTGT TGTATTGGGG AGGAAGGAGA GATTTYGAGA AGGATTAGTT TATTTTTTTA G
		[Btn]CCCCATACCTAATCACCCTTTAAC		S2: GGAGGAAGGAGAGATT	TYGAGAAGGA TTAGTTTATT TTTTTAGAAG ATAYGTGTGT ATTTTTTTGT TGTG
				S3: GAGAAGGATTAGTTTATTTTTTTA	GAAGATAYGT GTGTATTTTT TTGTTGTGYG GGTTTAGAAG GAGTTTAGYG TGTTAAAGGG TGATTAGGTA TGGG
Mlh1	-154 bp to -126 bp	AGTGTTGATTGGGTAGTATGAATG	55°C_45	GATATTAGGTTAGAAGGTAA	YGYGTTTGYG YGTTAAAGTT TAYGGTTYGT TTTTTTTATT GGTTTATTTT TGA
		[Btn]CCCTCAACTCTCAAAAATAAACCAATAA			
		[Btn]AACCCACAACTCAATAATCAAC			
Sfrp1	-72 bp to -54 bp	GGGTGGTAGAGTTTTGGATT	55°C_45	GTAGTTAATTAGTTT	YGYGTTGTYG GGAGTYGTTT TTTATAT
		[Btn]TCTAATAAACCCATCCTTCTAACC			
Gata4	-236 bp to -224 bp	GGAATTTTAGGGGAAGGAAAGTATTTAG	55°C_45	GTTTAGTTTTGTTTTTAGTGTAGTT	YGAYGTTTTT GYGGTTTGTT GTTTTGTTAA GGAT
		[Btn]AAATCACCTTCTCCTCTACC			

Supplementary Table 4. TaqMan assays and SYBR green primers for qRT-PCR

TaqMan assays (Thermo Fisher)			
**Gene**	**Assay ID**	**RefSeq**	**Exon Boundary**
p16	Mm00494449_m1	NM_009877.2	2-3
Ppara	Mm00440939_m1	NM_011144.6	7-8
Aldh1a1	Mm00657317_m1	NM_013467.3	5-6
Acaa1b	Mm00728805_s1	NM_146230.3	12-12
Cyp2c55	Mm00472168_m1	NM_028089.3	7-8
Nos2	Mm00440502_m1	NM_010927.3	21-22
Bst2	Mm01609165_g1	NM_198095.2	4-5
Ifitm3	Mm00847057_s1	NM_025378.2	2-2
Stat1	Mm01257286_m1	NM_009283.4	23-24
Ifng	Mm01168134_m1	NM_008337.3	3-4
Pdl1	Mm03048248_m1	NM_021893.3	5-6
Actb	mm00607939_s1	AK078935.1	6-6

SYBR green qRT-PCR primers		
**Gene**	**Forward sequence**	**Reverse sequence**
mLV30-2	CTCGCACCTTTTCGCGCTCG	GCGAGGGGATCATCATAACA
mMLV	CCAGACTTGATCCTGCTACA	CTCAGTCAGCCATCTCTGAC
mMuRRS	TTAGCCTCCTTCCGAGCTGA	GCCTCTGTCAGCCACCGTTT
p16	ATGGAGTCCGCTGCAGACAG	ATCGGGGTACGACCGAAAG
Actb	CATTGCTGACAGGATGCAGAAGG	TGCTGGAAGGTGGACAGTGAGG

Supplementary Table 5. Microsatellite markers for detection of MSI in mouse tumors

Marker	Repeat	Chromosome	GenBank accession	PCR size	Primer sequence
Bat-24	(A)24	7	U12235	98	Forward: 5'FAM-CATAGACCCAGTGCTCATCTTCGT<br>Reverse: gtttcttCATTCGGTGGAAAGCTCTGA
Bat-59	(A)59	16	NT_039624	189	Forward: 5'FAM-CATTCGGTGGAAAGCTCTGA<br>Reverse: gtttcttGGCTCACAACCATCCGTAACAAGA
Bat-67	(A)67	2	AL928868	266	Forward: 5'HEX-CCGACTGCTCTTCCGAAGGTC<br>Reverse: gtttcttTTGCCCATTTATCATCTAGTTCAT
D1Mit79	(CA)31	1	AF060887	150	Forward: 5'HEX-GACCTGGAAGTTGGAAACCA<br>Reverse: gtttcttTGATCCAGAACCTCTGCCTT
TG27	(TG)27	10	AC098712	136	Forward: 5'FAM-GGATCACTCGATGTACGGCTACTC<br>Reverse: gtttcttCCAGGCAGGCAAAGCATTTAT

- The PIGtailing of GTTTCTT on the 5' end of reverse primer was used as previously described (Brownstein et al. BioTechniques. 1996; 20(6): 1004-6).
